# Meta-Analysis of Gene Expression Signatures Defining the Epithelial to Mesenchymal Transition during Cancer Progression

**DOI:** 10.1371/journal.pone.0051136

**Published:** 2012-12-10

**Authors:** Christian J. Gröger, Markus Grubinger, Thomas Waldhör, Klemens Vierlinger, Wolfgang Mikulits

**Affiliations:** 1 Department of Medicine I, Division: Institute of Cancer Research, Comprehensive Cancer Center, Medical University of Vienna, Vienna, Austria; 2 Department of Epidemiology, Centre of Public Health, Medical University of Vienna, Vienna, Austria; 3 Austrian Institute of Technology, Vienna, Austria; Ghent University, Belgium

## Abstract

The epithelial to mesenchymal transition (EMT) represents a crucial event during cancer progression and dissemination. EMT is the conversion of carcinoma cells from an epithelial to a mesenchymal phenotype that associates with a higher cell motility as well as enhanced chemoresistance and cancer stemness. Notably, EMT has been increasingly recognized as an early event of metastasis. Numerous gene expression studies (GES) have been conducted to obtain transcriptome signatures and marker genes to understand the regulatory mechanisms underlying EMT. Yet, no meta-analysis considering the multitude of GES of EMT has been performed to comprehensively elaborate the core genes in this process. Here we report the meta-analysis of 18 independent and published GES of EMT which focused on different cell types and treatment modalities. Computational analysis revealed clustering of GES according to the type of treatment rather than to cell type. GES of EMT induced via transforming growth factor-β and tumor necrosis factor-α treatment yielded uniformly defined clusters while GES of models with alternative EMT induction clustered in a more complex fashion. In addition, we identified those up- and downregulated genes which were shared between the multitude of GES. This core gene list includes well known EMT markers as well as novel genes so far not described in this process. Furthermore, several genes of the EMT-core gene list significantly correlated with impaired pathological complete response in breast cancer patients. In conclusion, this meta-analysis provides a comprehensive survey of available EMT expression signatures and shows fundamental insights into the mechanisms that are governing carcinoma progression.

## Introduction

The epithelial to mesenchymal transition (EMT) has been originally described as an essential process of metazoan embryogenesis [1]. In the past decade, EMT has been realized as a critical event in carcinoma progression as epithelial tumor cells acquire a mesenchymal phenotype that allows them to detach from the primary tumor and to invade into the local tissue [2]. In general, polarized epithelial cells are organized by cell-cell junctions and cell-anchoring complexes to form apical and basolateral surfaces. In contrast, mesenchymal cells form irregularly shaped structures in the absence of tight adhesions to the neighboring cells and reduced cell contact to the substratum. Mesenchymal cells have an elongated shape compared to epithelia and display an anterior-posterior polarity that enables enhanced migration through reduced adhesion forces. While epithelial cells invade collectively in clusters, mesenchymal cells show individual cell movement that allows them to disseminate from bulk cells [3]. In addition, a partial EMT displaying different levels of E-cadherin expression has been observed that might still lead to collective cell invasion [4].

EMT has been classified into three subtypes [5]. Type 1 EMT is required for embryogenesis to provide gastrulation and formation of neural crest cells that differentiate into various cell types without systemic spreading. Type 2 EMT is involved in tissue regeneration and fibrosis of different organs such as the kidney, liver, lung and intestine leading to the accumulation of connective tissue. Type 3 EMT associates with a gain in malignancy of carcinoma cells. Neoplastic epithelial cells induced to undergo EMT are frequently localized at the invasive front of the primary tumor and initiate the cascade of tumor cell dissemination by local cell invasion which is followed by the entry into the vasculature. Notably, EMT represents a transient and reversible process that can lead to a mesenchymal to epithelial transition (MET) upon metastatic colonization [5], . Cycles of EMT and MET are assumed to be involved in metastasis formation at distal sites [3]. Yet, the molecular basis for the changes in epithelial plasticity by EMT and MET is still an open issue and its role in cancer patients is a matter of debate. Signaling molecules and inducers of type 3 EMT confer the resistance of cancer cells to apoptosis and oncogene-induced senescence as well as chemoresistance [6]. Recent findings indicate that EMT provides mesenchymal cells with stem cell features that enable carcinoma cells to generate metastasis at secondary sites [3]. These cancer stem cells, also termed cancer initiating cells, share phenotypic and functional characteristics with migratory embryonic cells displaying a mesenchymal phenotype [6].

Profiling of the transcriptome using microarrays has been widely used to elucidate the expression patterns during EMT under different conditions which revealed novel biomarkers and molecular mechanisms from single studies. A meta-analysis usually describes the combination of a large number of studies from different samples and tissues or the comparison of own data with published data [7], [8]. Recent progress in the establishment of gene expression datasets enables to identify new markers and relevant mechanisms which were underestimated in single studies but emerged from a meta-analysis. By now, a plethora of gene expression studies (GES) covering a wide variety of cell types undergoing EMT together with various modes of induction are available. Yet to our knowledge, no meta-analysis dealing with these EMT studies has been performed so far.

Changes in a biological system require a concerted alteration of gene expression sets. Bioinformatic enrichment analysis tools investigate gene expression sets for such changes. These tools examine the overrepresentation of gene sets in comparison to the whole genome, map an input list of genes to biological categories in online databases and statistically assess the overrepresentation of genes for each biological category or annotation such as Kyoto Encyclopedia of Genes and Genomes (KEGG) pathways and gene ontology (GO) terms [9]. The use of several single enrichment tools for the same input list and the consideration of only consistently enriched categories have been reported to be a very promising strategy [10], [11].

We gathered data from 18 published and independent GES of EMT and extracted gene lists of significantly up- and downregulated genes for cluster analysis. This approach revealed gene clusters according to treatment modalities rather than to cell type. We subsequently extracted an EMT-core list consisting of 130 genes with official gene symbols and names which was further investigated by enrichment analysis with several single enrichment tools. Notably, selected genes from the EMT-core list significantly correlated with impaired pathological complete response (pCR) in breast cancer patients. This analysis proposes that the EMT-core gene list is relevant for the recognition of the molecular mechanisms of EMT. In addition, the cluster analysis shows novel insights into the relationships of EMT processes across different cell types and induction modes.

## Results

### Data collection of gene expression studies (GES)

To assess the similarities between published GES and define a core gene list of human EMT, we analyzed 18 independent GES of EMT. These 18 independent and published GES consisted of 24 datasets in total (Table 1). Several authors reported EMT kinetics of different cell types or dose-dependent effects of EMT inducers within single studies. Nevertheless, only the particular testing point showing the strongest effect or EMT phenotype, as reported by the authors, has been selected. Takahashi *et al.* published two related GES, of which one consisted of two datasets, resulting in three datasets of one independent study [12]. Taube *et al.* reported 5 datasets published within one GES with similar expression patterns and different modes of EMT induction [13]. Processed data (normalized and generally logarithmized data) were downloaded from the Gene expression Omnibus (GEO) and ArrayExpress (AE) databases and annotated with BioConductor and NetAffx. Numerous GES, available on GEO and AE, were excluded as they either did not provide processed data or did not contain replicates or have not been published. Due to the variety of microarray formats as well as different normalization and filtering methods used in the literature, we used processed instead of raw data in order to maintain the quality criteria applied by the authors during the data preprocessing. Two-tailed Student's *t*-test was used to compute p-values. Significantly up- and downregulated genes were selected to meet a fold change greater than 2 or lower than 0.5 and a p-value below 0.05.

**Table 1:** Gene expression studies of EMT used for meta-analysis.

First author	Acc.	Ref.	Cell type	Cell origin	Treatment modality	Platform	Samples*
Ke	E-TABM-949	[28]	EP156T/EPT2	Prostate	high cell density^a^	Agil WHG 4×44K G4112F	2
Andarawewa	GSE8240	[61]	MCF10A	Breast	TGF-β+irradiation^a^	Affy HTU133A	3
Takahashi^c^	GSE12548/GSE15205	[12]	ARPE19	Retinal pigment	TGF-β+TNF-α^a^/TGF-β or TNF-α^a^	Affy U133Plus2	3
Tay	GSE13759	[62]	HCT116/E1	Colon	serial transplantation^b^	Affy U133A	3
Drake	GSE14405	[63]	PC-3/TEM4-18	Prostate	transendothelial migration^a^	Affy U133Plus2	2
Hwang	GSE14773	[19]	CRC	Colon	spheroid formation^a^	Affy U133Plus2	2
Sartor	GSE17708	[64]	A549	Lung	TGF-β^a^	Affy U133Plus2	3
Papageorgis	GSE18070	[65]	MCF10CA1h	Breast	H-Ras+carcinoma^b^	Affy U133Plus2	3
Hills	GSE20247	[66]	HK2	Kidney	TGF-β+Cpep^a^	Illum HWG-6 v3.0	3
Leshem	GSE22010	[67]	PrEC-hTERT	Prostate	AR+T/ERG^a^	Affy HG 1.0 ST	4
Micalizzi	GSE23655	[20]	MCF7	Breast	Six1 vector^a^	Affy HTU133A	6
Maupin	GSE23952	[68]	Panc-1	Pancreas	TGF-β^a^	Affy U133Plus2	3
Taube^d^	GSE24202	[13]	HMLE	Breast	TGF-β1; Snail1, Twist, Gsc vectors; siRNA against E-Cadherin a	Affy HTU133A	3
Baniwal	GSE24261	[21]	PCa C4-2B/Rx2dox	Prostate	Runx2 vector^a^	Illum HR-8 v3.0	4
van Zijl	GSE26391	[26]	3p/3sp	Liver	tumor cell recovery^b^	Affy HG 1.0 ST	2
Ohashi	GSE27424	[29]	EPC2-hTERT	Esophagus	Notch3 knock-down (shRNA)^a^	Affy U133Plus2	3
Hesling	GSE28448	[69]	HMEC-TR	Breast	TGF-β+siRNA against TIFγ^a^	Affy U133Plus2	2
Wang	GSE28799	[70]	OVCAR-3	Ovary	spheroid formation^a^	Affy U133Plus2	3

* , lowest number of samples per class (control or test subject).

a , *in vitro*;

b , *in vivo*;

c , consists of two studies with three datasets in total; d, consists of five datasets.

Abbreviations: Affy, Affymetrix; Agil, Agilent; AR, androgen receptor; Illum, Illumina; sh, small hairpin; si, small interfering; T/ERG, TMPRSS2/ERG; TGF, transforming growth factor; TNF, tumor necrosis factor.

### GES cluster analysis

**Figure 1:**
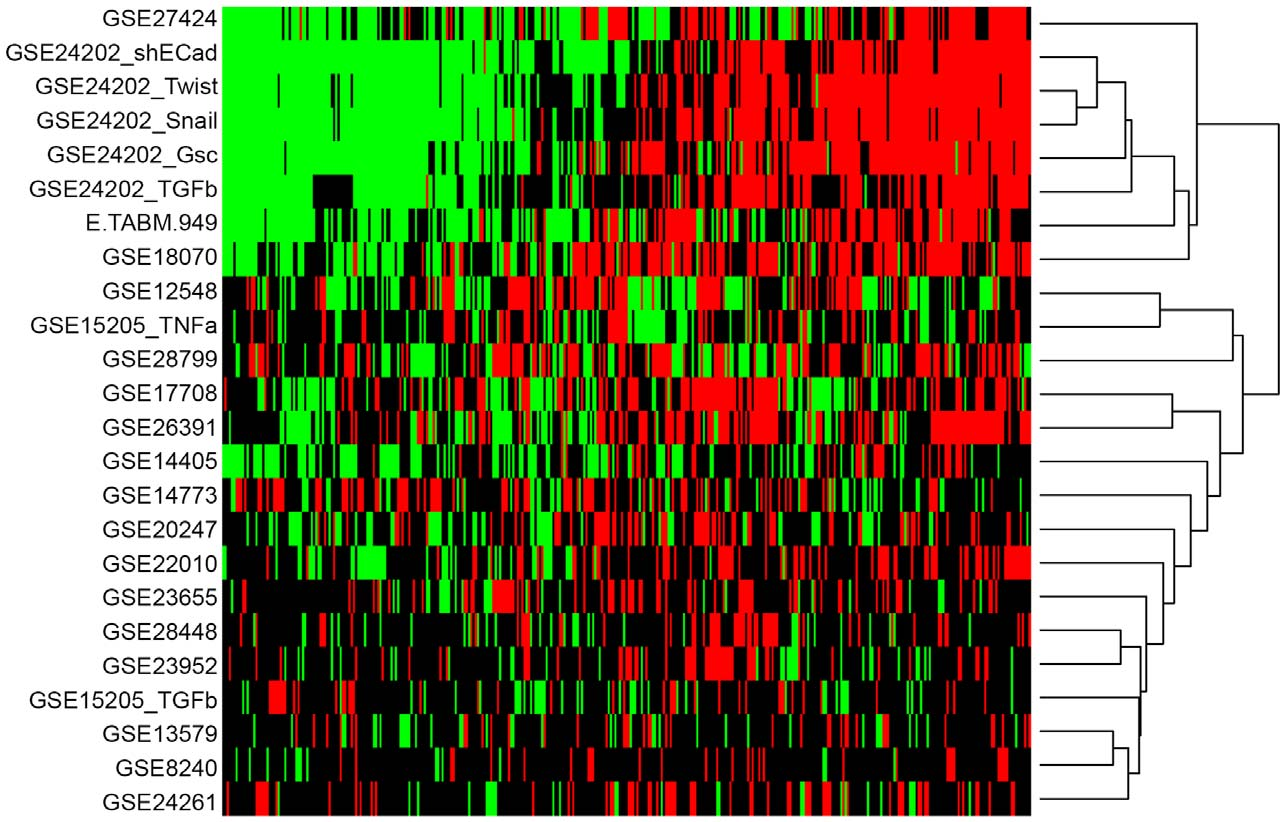
Cluster analysis of genes shared between at least 10 GES datasets shows distinguishable and significant clusters. Genes shared between at least 10 out of 24 datasets were used for Manhattan hierarchical clustering. The type of regulation within a particular study was visualized via heatmap. Columns: genes shared between at least 10 datasets (n = 365); rows: analyzed GES (24 datasets in total); green: downregulated genes; red: upregulated genes; black: genes not regulated. GSE: Gene expression omnibus (GEO) series record; E.TABM: ArrayExpress (AE) series record; TGF, transforming growth factor; TNF, tumor necrosis factor.

**Figure 2:**
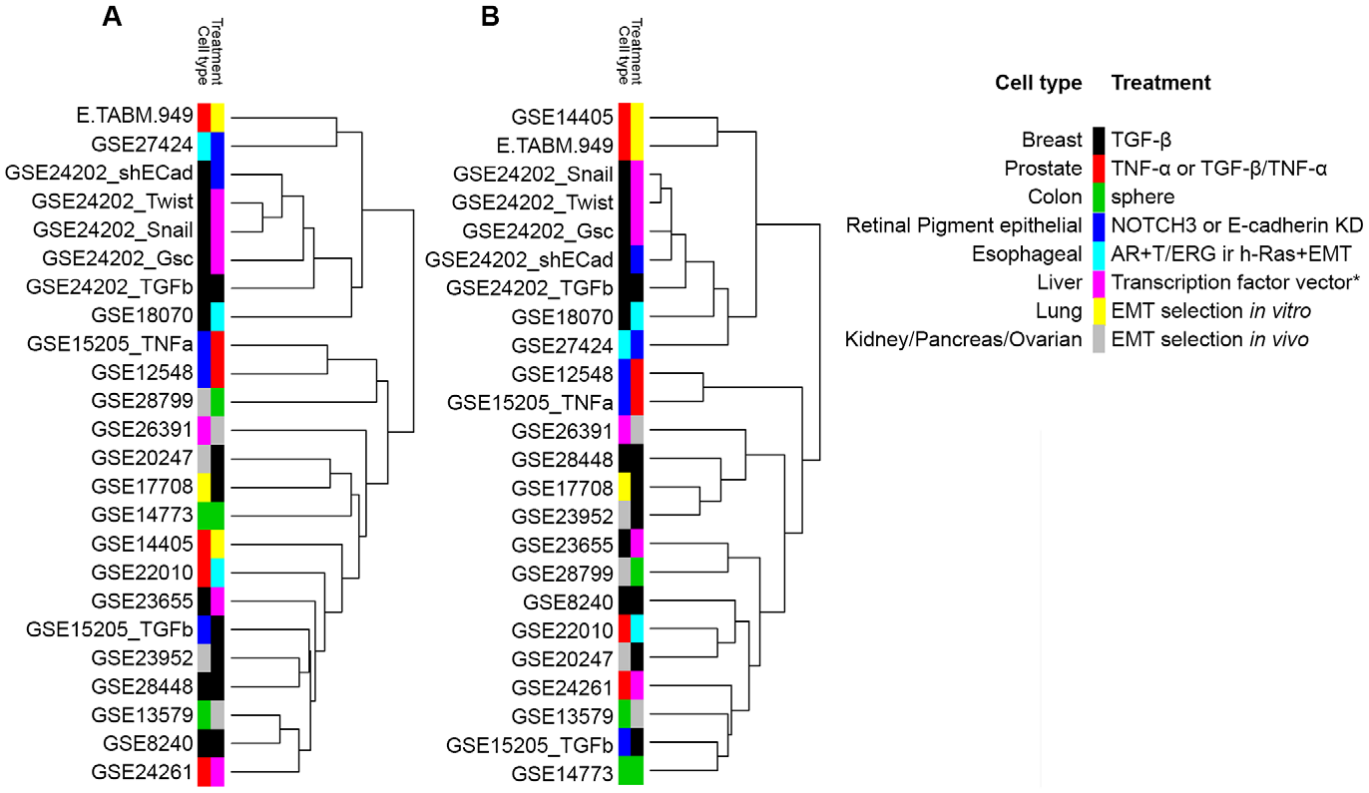
Gene expression studies cluster according to the mode of EMT initiation rather than to cell type. The cell type and treatment modality of EMT was annotated and revealed clustering according to the mode of EMT induction. The clustering persisted when genes shared between at least 14 GES datasets were used for the analysis. (A) Hierarchical clustering of 365 genes shared between at least 10 datasets. (B) Hierarchical clustering of 41 genes shared between at least 14 datasets. The legend indicates cell type and treatment modality (right panel). *, Transcription factor vectors: Runx2, Six1, Snail, Twist and Goosecoid. GSE: Gene expression omnibus (GEO) series record; E.TABM: ArrayExpress (AE) series record; TGF, transforming growth factor; TNF, tumor necrosis factor.

We generated a matrix containing gene symbols across the analyzed GES (n = 14,113) that are all uniquely reported. Significantly up- and downregulated genes of each GES were transferred into the matrix according to their type of regulation. Upregulated genes were labeled with 1, downregulated genes with −1 and not differentially regulated genes with 0 (Table S1). This data distribution consisted of 88.22% not differentially regulated genes and 11.78% up- or downregulated genes and is significantly different to a binomial distribution with those parameters (p<0.0001). In order to determine a cutoff for the number of GES sharing a particular gene used for cluster analysis, the binomial distribution function provided by R as well as the preliminary hierarchical clustering results of each cutoff option were analyzed (data not shown). From this we decided to investigate the clustering of genes shared between at least 10 datasets (n = 365; p<0.0001; Figure 1). In addition, this analysis showed clusters of GES according to the mode of EMT stimulus rather than to cell type (Figure 2A). Interestingly, a more stringent clustering of genes shared between at least 14 of the analyzed GES datasets provided similar clusters, despite the fact that this list contains only 41 genes (Figure 2B and Figure S1).

### Generation of the EMT-core gene list

Based on the cluster analysis of the GES, we aimed to define a meaningful EMT-core gene list which describes the majority of the involved genes across the analyzed GES. The cluster analysis of the genes shared between at least 10 datasets contained 365 genes (Table S2). However, it does not show whether a gene is up- or downregulated across different GES. Therefore, the list was filtered to keep only genes which were either up- or downregulated in at least 10 of the GES datasets. The resulting list contained 130 genes of which 67 are up- and 63 are downregulated (Table 2 and Table S3). This selection of genes could be further classified into five categories ((i) cell adhesion and migration, (ii) development, cell differentiation and proliferation, (iii) angiogenesis and wound healing, (iv) metabolism, (v) others or unclassified) according to single enrichment analysis as described below. Several genes were also present in more than one of those categories (Table S3). In conclusion, this resulting EMT-core gene list contains 130 genes which were derived from a multitude of cell types and EMT initiation methods.

**Table 2:** EMT-core list of 130 genes shared between at least 10 GES datasets.

	Upregulated	Downregulated
**Cell adhesion and migration**	ADAM12, CDH11, CDH2, COL1A1, COL3A1, COL5A1, COL6A1, COL6A3, CTGF, CYP1B1, DLC1, FBLN1, FBLN5, FGF2, FGFR1, FN1, HAS2, LUM, MMP2, MYL9, NID2, NR2F1, NRP1, PLAT, PPAP2B, PRKCA, RECK, SERPINE1, SERPINE2, SPOCK1, TGM2, TNFAIP6, TPM1, VCAN, WNT5A	CD24, CDH1, CXADR, CXCL16, DSG3, ELF3, EPCAM, EPHA, JUP, MPZL2, OVOL2, PLXNB1, S100P, SLC7A5, SYK
**Development, cell differentiation and proliferation**	CDKN2C, EMP3, FBN1, IGFBP3, IL1R1, LTBP1, MME, PMP22, PTGER2, PTX3, SRGN, SULF1, SYNE1, TAGLN, TUBA1A, VIM, ZEB1	ABLIM1, ADRB2, ALDH1A3, ANK3, BIK CA2, CTSL2, FGFR2, FGFR3, FST, GJB3, IFI30, IL18, KLK7, KRT15, KRT17, LSR, MAP7, MBP, OCLN, PKP2, PPL, PRSS8, RAPGEF5, SPINT1
**Angiogenesis and wound healing**	DCN, LOX, TFPI	*no gene with a major classification* *
**Metabolism**	ABCA1, GALNT10, SLC22A4	GPX3, SLC27A2, SMPDL3B, SORL1, ST6GALNAC2
**Others or unclassified**	C5orf13, CDK14, EML1, FSTL1, LTBP2, MAP1B, RGS4, SYT11, TMEM158	AGR2, C10orf10, CDS1, FAM169A, FXYD3, KLK10, LAD1, MTUS1, PLS1, PRRG4, RHOD, SERPINB1, SLPI, TMEM30B, TPD52L1, TSPAN1, ZHX2, ZNF165

Categories have been chosen according to the GO classifications of the enrichment tools. Genes may be present in more than one category.

* see Table S3 for more information.

### Consistently enriched KEGG pathway and GO term analysis of the EMT-core gene list

**Figure 3:**
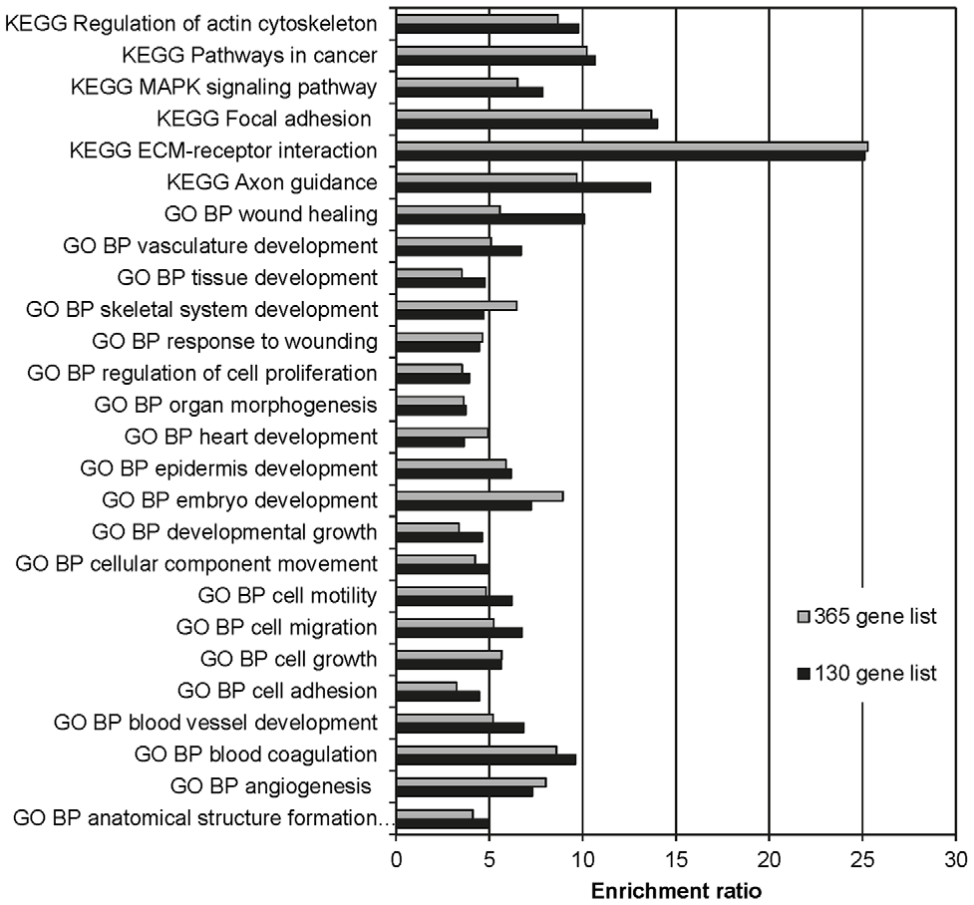
The 130 genes EMT-core list and the 365 genes list exhibit comparable enrichment ratios of GO biological processes and KEGG pathways. The enrichment ratio is the number of observed genes divided by the number of expected genes for a given term or pathway. Enrichment ratios were obtained from WebGestalt or calculated with data from FatiGO. GO, gene ontology; BP, biological process; KEGG, Kyoto encyclopedia of genes and genomes.

To further analyze the EMT-core list consisting of 130 genes, a rigorous single enrichment analysis combined with stringent selection criteria was performed. First, an enriched KEGG pathway or GO term had to contain at least 5 genes from the input list and a p-value below 0.05 to be considered significant. An enumeration of significantly enriched terms and pathways is shown in Table 3. Second, a significantly enriched KEGG pathway or GO term had to be observed in at least 4 out of 5 used bioinformatic tools. Third, a consistently enriched KEGG pathway or GO term had to be identified in both the EMT-core gene list and the 365 gene list. Using these criteria, we obtained 6 KEGG pathways, 20 GO biological processes and 15 GO molecular functions consistently enriched in both lists (Table 4). The KEGG pathways consisted of the MAPK signaling pathway, axon guidance, focal adhesion, ECM-receptor interaction, regulation of actin cytoskeleton and pathways in cancer. The GO biological processes could be grouped into processes involved in tissue development, wound healing, cell migration or cell proliferation. The GO molecular functions consisted of ECM and cytoskeleton constituents, peptidase inhibitors and the binding of collagen, growth factors, heparin and integrin. As expected, the list with 365 genes comprised all significantly enriched pathways and GO terms from the 130 genes EMT-core list except for 2 GO biological processes (ECM organization and lung development). Several more KEGG pathways, GO biological processes and molecular functions could be identified in the list with 365 genes (Table 3 and 4). All these pathways, biological processes and molecular functions are well known to be involved in EMT [5], [14]–[16], and thus confirm the integrity of our EMT-core gene list. In addition, both the EMT-core list and the list with 365 genes display comparable enrichment ratios of KEGG pathways and GO biological processes (Figure 3) as well as GO molecular functions (Figure S2). Therefore, the list containing 365 genes may be considered as an amelioration of the EMT-core list by containing additional genes that might have an ambiguous role in EMT. In summary, our EMT-core list of 130 genes and its amelioration containing 365 genes show strong enrichment of EMT-relevant processes.

**Table 3:** Number of enriched terms and pathways in all lists detected by the enrichment tools.

Tool	130 gene list			365 gene list			GSE13195 core list			GSE24202 core list		
	BP	MF	KEGG	BP	MF	KEGG	BP	MF	KEGG	BP	MF	KEGG
ConsensusPathDB	305	31	9	558	61	31	62	10	6	247	34	8
FatiGO	178	28	9	452	72	36	0	0	2	172	28	10
GeneCodis	34	16	8	155	45	46	59	17	4	240	48	7
ToppFun	241	21	1	610	45	5	0	0	1	127	14	0
WebGestalt	40	28	6	40	40	37	5	4	4	40	30	8

The numbers of enriched terms and pathways found by the particular enrichment tools are displayed. BP, GO biological process; MF, GO molecular function; KEGG, KEGG pathway. GSE13195 core list of Choi *et al.*, GSE24202 core list of Taube *et al.* [13], [39].

**Table 4:** Consistently enriched GO terms and KEGG pathways and their occurrence in the analyzed gene lists.

Term ID	Category	Term size*	130 gene list		365 gene list		GSE13915 core list		GSE24202 core list	
			Tools	Genes	Tools	Genes	Tools	Genes	Tools	Genes
**GO biological process**										
GO:0048646	anatomical structure formation involved in morphogenesis	390	4	24	4	62	0	-	4	22
GO:0001525	angiogenesis	189	4	16	4	38	0	-	4	14
GO:0007596	blood coagulation	182	4	13	4	29	0	-	3	13
GO:0001568	blood vessel development	288	4	25	5	54	0	-	5	20
GO:0007155	cell adhesion	953	5	36	5	76	2	19	5	41
GO:0016049	cell growth	226	4	13	4	34	0	-	4	14
GO:0016477	cell migration	405	5	32	5	67	1	13	5	35
GO:0048870	cell motility	484	4	33	4	69	1	13	5	35
GO:0006928	cellular component movement	666	4	36	5	73	1	16	5	41
GO:0009790	embryo development	619	4	18	4	46	0	-	3	20
GO:0008544	epidermis development	218	5	16	4	32	2	6	5	26
GO:0007507	heart development	230	5	15	4	28	1	6	3	10
GO:0009887	organ morphogenesis	800	5	21	5	54	0	-	5	34
GO:0042127	regulation of cell proliferation	823	4	28	5	81	1	14	5	37
GO:0050793	regulation of developmental process	1005	4	34	4	88	0	-	4	32
GO:0009611	response to wounding	776	5	31	5	85	0	-	4	34
GO:0001501	skeletal system development	394	4	14	4	35	0	-	5	20
GO:0009888	tissue development	808	4	38	4	93	1	12	5	52
GO:0001944	vasculature development	294	4	25	4	56	0	-	5	20
GO:0042060	wound healing	270	4	20	5	50	0	-	3	19
**GO molecular function**										
GO:0005509	calcium ion binding	1033	4	22	4	55	0	-	4	34
GO:0030246	carbohydrate binding	380	4	15	4	29	1	7	4	14
GO:0005518	collagen binding	40	4	5	5	12	0	-	0	-
GO:0004866	endopeptidase inhibitor activity	179	4	9	4	19	0	-	4	9
GO:0004857	enzyme inhibitor activity	327	4	10	4	26	2	8	4	13
GO:0005201	ECM constituent	105	5	7	5	12	0	-	4	7
GO:0005539	glycosaminoglycan binding	146	4	13	5	24	0	-	5	10
GO:0019838	growth factor binding	127	4	13	4	26	0	-	5	14
GO:0008201	heparin binding	108	4	9	4	17	0	-	3	7
GO:0005178	integrin binding	57	4	6	5	9	0	-	4	7
GO:0030414	peptidase inhibitor activity	192	5	9	5	20	0	-	4	9
GO:0030247	polysaccharide binding	165	4	14	4	27	0	-	5	13
GO:0032403	protein complex binding	199	4	11	4	20	0	-	2	8
GO:0004867	serine-type endopeptidase inhibitor activity	118	4	9	4	14	0	-	3	7
GO:0005200	structural constituent of cytoskeleton	92	5	8	5	10	0	-	5	13
**KEGG pathway**										
map04360	axon guidance	126	4	6	4	11	0	-	4	6
map04512	ECM-receptor interaction	92	5	7	5	18	0	-	1	5
map04510	focal adhesion	207	4	9	5	23	0	-	3	8
map04010	MAPK signaling pathway	289	3	7	4	15	0	-	0	-
map05200	pathways in cancer	329	4	11	5	28	1	5	2	8
map04810	regulation of actin cytoskeleton	209	4	7	4	16	0	-	2	6

* According to FatiGO category size in genome.

The maximum number of genes from the category present in the input list is displayed. ID, identity; GO, gene ontology; KEGG, Kyoto encyclopedia of genes and genomes. GSE13195 core list of Choi *et al.*, GSE24202 core list of Taube *et al.* [13], [39].

### Clinical relevance of the EMT-core gene list

The EMT-core gene list contains several genes with yet unidentified roles in cancer progression and/or EMT. We aimed to investigate the clinical relevance of this selection of genes. Therefore, we correlated their expression with overall survival of patients suffering from squamous cell lung carcinomas (SCC) [17] and pathological complete response (pCR) of breast cancer patients [18]. From the downregulated genes of the EMT-core gene list, low FXYD3 expression showed a trend to poor overall survival of SCC patients (p = 0.17) and low expression of LAD1 (p = 0.00074), SLC7A5 (p = 0.0093) and SLPI (p = 0.043) significantly correlated with worse pCR of breast cancer patients. From the upregulated genes of the EMT-core gene list, high PTX3 expression tends to poor overall survival of SCC patients (p = 0.16) and high expression of NID2 (p = 0.0091), SPOCK1 (p = 0.038) and SULF1 (p = 0.00029) significantly correlated with impaired pCR of breast cancer patients. These correlations demonstrate that the comparison of different data sets is a powerful tool to identify novel relevant target genes that do not emerge from single studies.

## Discussion

Over the past decade a considerable number of GES that deal with EMT have been accumulating in the literature. These cover a variety of cell types which display EMT and include different modes of EMT induction. So far, these resources have only been partially used to compare single findings with those in the literature [8], [19], [20]. To our knowledge, no attempt has been made to investigate the majority of the independent GES of EMT for their relations to each other. Although we are aware that gene expression data of EMT are not complete, we analyzed the currently available GES to generate an EMT-core list of genes altered most frequently during the EMT process, as depicted in the flow chart (Figure S3).

Cluster analysis of genes shared between at least 10 GES datasets revealed clusters of GES with the same or a similar treatment type. The GES in which EMT was induced by TNF-α either alone or in combination with TGF-β, by TGF-β alone or by different transcription factors consistently grouped together. These clusters persisted when genes shared between at least 14 datasets were used for cluster analysis. A clear clustering of different types of EMT induction, however, would have only been possible if an adequate number of GES on each of these EMT initiation methods existed. Since several treatment modalities are only represented once in the literature, such GES cluster to their most related treatment type.

One cluster predominantly emerged from GES of TGF-β-induced EMT which consisted of 13 datasets. Interestingly, the cluster includes the exogenous expression of Six1 (Micalizzi *et al*; GSE23655; [20]) which has been shown to enhance tumor-promoting TGF-β signaling, and Runx2 (Baniwal *et al*; GSE24261; [21]) that acts downstream of TGF-β signaling [22]–[25]. Hence, this supports the clustering of these studies together with others using TGF-β as EMT initiator. The study by van Zijl *et al.* (GSE26391; [26]) described the analysis of epithelial and mesenchymal hepatocellular carcinoma cells derived from the same tumor patient. The clustering of this study along with other studies with TGF-β-induced EMT suggests an involvement of TGF-β signaling during the establishment of the mesenchymal cell line.

The cluster of GES with TNF-α as EMT inducer contained the study by Takahashi *et al.* which analyzed the ARPE19 cell line treated with either TNF-α alone (GSE15205_TNFa), TNF-α together with TGF-β (GSE12548) or TGF-β alone (GSE15205_TGFb) in order to induce EMT [12]. The two datasets with TNF-α treatment formed a consistent cluster. However, the third dataset which was obtained from the exclusive treatment with TGF-β clustered to other GES describing EMT initiation by TGF-β. Hence, these data suggest a stronger impact of the EMT stimulus on the clustering rather than the cell type.

One cluster consisted mainly of the datasets from Taube *et al.* (GSE24202; [13]) who reported the induction of EMT in HMLE cells using overexpression of Twist, Snail, Goosecoid and TGF-β as well as the knockdown of E-cadherin. Consistent with the data reported by Taube *et al*, the datasets from Snail- and Twist-induced EMT were the most similar within this cluster. This finding is concordant with the fact that Twist is a direct target of Snail [27]. The high number of datasets in this study might lead to an overrepresentation within the cluster analysis. Furthermore, the use of the same cell line as well as transcription factors with similar targets such as Twist and Snail might lead to a high level of similarity within the datasets of this particular study.

The cluster comprising of Ke *et al.* (E-TABM-949; [28]) who utilized high cell density culturing of EPT2 cells and Ohashi *et al.* (GSE27424; [29]) who described a NOTCH3 knock-down in EPC2 cells displays a low level of relation to other clusters due to the unique types of EMT induction. It appears likely that on the one hand these GES form a cluster due to the lack of relationship to the other clusters. On the other hand, it might also suggest a relation of their types of EMT initiation as well.

We found a variety of well-known markers of EMT upregulated in our EMT-core gene list such as CDH2, CDH11, COL1A1, COL3A1, FBLN5, FN1, HAS2, LOX, MMP2, PLAT, SERPINE1, VIM, WNT5A and ZEB1 [15], [30], [31]. Furthermore, we detected downregulated genes reported to be reduced in EMT such as ANK3, CDH1, CXADR, PRSS8 and SYK [15], [32]–[34], several downregulated epithelial cell markers such as EPCAM, JUP, KRT15, KRT17, OCLN, PKP2 and PPL [5], [15] and a number of downregulated tumor suppressors such as KLK10, MTUS1, OAS1 and SERPINB1 [35]–[38]. Together, these genes provide a solid verification of our EMT-core gene list. Besides those genes confirming the integrity of our gene list, however, genes with unknown functions as well as an unknown or unclear relation to cancer and/or EMT emerged which are novel candidates for further investigation. Upregulated genes include MAP1B, NID2, PTX3, SPOCK1, SULF1, TAGLN and TMEM158 while downregulated genes comprised ABLIM1, LAD1, FAM169A, FXYD3, SLC7A5, SLPI, TMEM30B and TPD52L1.

Two meta-analyses of EMT in breast cancer considering different cell lines or types of EMT induction have been reported. These have identified EMT-core gene lists with 200 and 251 genes [13], [39], however, overlapping with approximately 10% only. Our EMT-core list containing 130 genes shows a poor overlap of 7% with the list of Choi *et al.* [39] but an overlap of 55% with Taube *et al.* [13]. Both the lists by Choi *et al.* and Taube *et al.* contain unmapped identifiers (IDs) such as array IDs, expressed sequence tags and locus IDs. We used consistently enriched pathway analysis to further investigate these gene lists. Notably, our EMT-core list displayed more enriched KEGG pathways and GO terms than the gene lists of Choi *et al.* and Taube *et al.* (Table 3 and 4). Upon reducing the stringency of analysis to two genes within an enriched category, the enrichment for the list of Choi *et al.* did not improve whereas nearly all KEGG pathways and GO terms enriched in our EMT-core list could be observed in the list of Taube *et al.* (data not shown, Table 4).

The EMT-core list contains several genes with unknown functions and relations to cancer and/or EMT. We were able to show that FXYD3 and PTX3 expression is associated with poor overall patient survival in SCC patients and LAD1, SLC7A5, SLPI, NID2, SPOCK1 and SULF1 correlated significantly with impaired pCR in breast cancer patients. FXYD3 has been shown to be involved in tumor cell proliferation and to be downregulated by TGF-β signaling [40], [41]. PTX3 has been reported to be a lung cancer biomarker [42]. NID2 has been shown to be elevated during phorbol 12-myristate 13-acetate-induced invasion of several human tumor cell lines and as a potential tumor biomarker [43], [44]. SPOCK1 has been reported to be involved in neuronal attachment and matrix metalloproteinase activation [45], [46]. SULF1 has been shown to be a potential biomarker for gastric cancer which can be induced by TGF-β1 [47], [48]. LAD1 is an adaptor protein involved in ERK5 and JNK pathways [49]. SLPI has been reported to act anti-tumorigenic for certain tumors as well as to promote migration and invasion in others [50]–[52]. Hence, these genes seem to be promising candidates for further investigation. Taken together, we propose that the EMT-core list of 130 genes is highly relevant for EMT and the cluster analysis represents a useful overview on the relationships of currently available GES of EMT.

## Materials and Methods

### Data collection and annotation

Processed microarray data were downloaded from the websites of GEO (available: http://www.ncbi.nlm.nih.gov/geo/) and AE (available: http://www.ebi.ac.uk/arrayexpress/) by using “EMT” as keyword for published GES until February 2012. The downloaded GES were annotated to retrieve official gene symbols, EntrezID and gene names using BioConductor 2.9 (available: http://www.bioconductor.org/; accessed: 2012 Jan 02) [53] and the online tool NetAffx (available: http://www.affymetrix.com/analysis/index.affx; accessed: 2012 June 25). BioConductor was used within the R environment [54]. Annotated data was imported to MS-Excel 2010 and log2 transformed. Subsequently, fold changes and p-values using two-sided Student's *t*-test were calculated. Significantly up- and downregulated genes were selected and separated from each other when showing a fold change greater than 2 or below 0.5 and a p-value below 0.05. Upregulated genes were ordered from highest to lowest fold change. Vice versa, downregulated genes were arranged from lowest to highest fold change. Duplicates were removed afterwards. Gene symbols have been used for further analysis and will be referred to as genes.

### Cluster analysis

The up- and downregulated genes from each study were summarized, ordered and duplicates were removed to obtain a list of all uniquely reported genes across all studies. Upregulated genes were labeled with 1 and downregulated genes were labeled with −1. Genes that were not significantly deregulated within a GES and genes which were found to be both up- and downregulated within a study were labeled with 0. The distribution of the observed number of up- and downregulated genes was tested against a binomial distribution with parameter p = 11.78% by means of a chi-squared test. We calculated the possibilities of drawing each cutoff option for cluster analysis (>1, >2, >3, and so forth) by chance with the binomial distribution function provided by R (probability = 11.78%). The possibilities to draw each cutoff option by chance were compared to preliminary cluster analyses of each cutoff option in order to determine a suitable cutoff. The clustering was performed in BioConductor 2.9 embedded in R 2.14.1 (64 bit) with the packages gdata [55], gplots [56] and heatmap.plus [57] using hierarchical heatmap clustering with Manhattan distance function.

### Consistently enrichment of KEGG pathways and GO terms

The gene lists were analyzed using five different bioinformatic enrichment tools. A comprehensive overview of the used tools and their characteristics is shown in Table S4. The tools FatiGO and GeneCodis were used on the Babelomics 4 platform [58], which provided access to both programs at once. The selection criteria for significantly enriched pathways were a p-value or FDR below 0.05 and a minimum of 5 genes of the input list within an enriched category. Furthermore, consistently enriched GO terms and KEGG pathways were identified in at least 4 of 5 programs in both the EMT-core gene list and the 365 gene list. Enrichment ratios (number of observed genes divided by the number of expected genes for a GO or KEGG category) have been obtained by WebGestalt, or alternatively, have been calculated as described by Zhang *et al.* with the data from FatiGO [59].

### Correlation of the EMT-core list with clinical data

Microarray and clinical data for patients with squamous cell lung carcinomas (n = 130) reported by Raponi *et al.* [17] with the accession GDS2373 were downloaded from GEO. Microarray and clinical data for breast cancer patients (n = 133) reported by Hess *et al.* [18] were downloaded from the MD Anderson Cancer Center website (available: http://bioinformatics.mdanderson.org/pubdata.html; accessed 2012 Sep 07). Patients were divided into high and low expressing groups for selected genes within the EMT-core list. The p-values were computed using two-sided Student's *t*-test. Survival analysis for the data by Raponi *et al.* was performed with the chi-squared test of equality using the survival package in R [60]. P-values below 0.05 were considered significant.

## Supporting Information

Figure S1 **Cluster analysis of genes shared between at least 14 GES datasets shows persistent and distinct clusters.**
(PDF)

Figure S2 **The 130 genes EMT-core list and the 365 genes list exhibit comparable enrichment ratios of GO molecular functions.**
(PDF)

Figure S3 **Flow chart depicting the generation of the EMT-core gene list.**
(PDF)

Table S1 Matrix containing significantly up- and downregulated genes across the analyzed GES datasets.
(XLS)

Table S2 List of 365 genes significantly regulated in at least 10 GES datasets.
(DOC)

Table S3 EMT-core gene list of 130 up- or downregulated genes shared between at least 10 GES datasets.
(DOC)

Table S4 Enrichment tools used in this study and their properties.
(DOC)

## References

[1] Hay ED (1995) An overview of epithelio-mesenchymal transformation. Acta Anat (Basel) 154: 8–20. 8714286 10.1159/000147748

[2] Hay ED (2005) The mesenchymal cell, its role in the embryo, and the remarkable signaling mechanisms that create it. Dev Dyn 233: 706–720. 15937929 10.1002/dvdy.20345

[3] van Zijl F, Krupitza G, Mikulits W (2011) Initial steps of metastasis: cell invasion and endothelial transmigration. Mutat Res 728: 23–34. 21605699 10.1016/j.mrrev.2011.05.002 PMC4028085

[4] Friedl P, Alexander S (2011) Cancer invasion and the microenvironment: plasticity and reciprocity. Cell 147: 992–1009. 22118458 10.1016/j.cell.2011.11.016

[5] Kalluri R, Weinberg RA (2009) The basics of epithelial-mesenchymal transition. J Clin Invest 119: 1420–1428. 19487818 10.1172/JCI39104 PMC2689101

[6] Thiery JP, Acloque H, Huang RY, Nieto MA (2009) Epithelial-mesenchymal transitions in development and disease. Cell 139: 871–890. 19945376 10.1016/j.cell.2009.11.007

[7] Loboda A, Nebozhyn MV, Watters JW, Buser CA, Shaw PM, et al (2011) EMT is the dominant program in human colon cancer. BMC Med Genomics 4: 9. 21251323 10.1186/1755-8794-4-9 PMC3032646

[8] Tobin NP, Sims AH, Lundgren KL, Lehn S, Landberg G (2011) Cyclin D1, Id1 and EMT in breast cancer. BMC Cancer 11: 417. 21955753 10.1186/1471-2407-11-417 PMC3192789

[9] Huang da W, Sherman BT, Lempicki RA (2009) Bioinformatics enrichment tools: paths toward the comprehensive functional analysis of large gene lists. Nucleic Acids Res 37: 1–13. 19033363 10.1093/nar/gkn923 PMC2615629

[10] Rhee SY, Wood V, Dolinski K, Draghici S (2008) Use and misuse of the gene ontology annotations. Nat Rev Genet 9: 509–515. 18475267 10.1038/nrg2363

[11] Lascorz J, Chen B, Hemminki K, Forsti A (2011) Consensus pathways implicated in prognosis of colorectal cancer identified through systematic enrichment analysis of gene expression profiling studies. PLoS One 6: e18867. 21541025 10.1371/journal.pone.0018867 PMC3081819

[12] Takahashi E, Nagano O, Ishimoto T, Yae T, Suzuki Y, et al (2010) Tumor necrosis factor-alpha regulates transforming growth factor-beta-dependent epithelial-mesenchymal transition by promoting hyaluronan-CD44-moesin interaction. J Biol Chem 285: 4060–4073. 19965872 10.1074/jbc.M109.056523 PMC2823547

[13] Taube JH, Herschkowitz JI, Komurov K, Zhou AY, Gupta S, et al (2010) Core epithelial-to-mesenchymal transition interactome gene-expression signature is associated with claudin-low and metaplastic breast cancer subtypes. Proc Natl Acad Sci U S A 107: 15449–15454. 20713713 10.1073/pnas.1004900107 PMC2932589

[14] Yilmaz M, Christofori G (2010) Mechanisms of motility in metastasizing cells. Mol Cancer Res 8: 629–642. 20460404 10.1158/1541-7786.MCR-10-0139

[15] Zeisberg M, Neilson EG (2009) Biomarkers for epithelial-mesenchymal transitions. J Clin Invest 119: 1429–1437. 19487819 10.1172/JCI36183 PMC2689132

[16] Hanahan D, Weinberg RA (2011) Hallmarks of cancer: the next generation. Cell 144: 646–674. 21376230 10.1016/j.cell.2011.02.013

[17] Raponi M, Zhang Y, Yu J, Chen G, Lee G, et al (2006) Gene expression signatures for predicting prognosis of squamous cell and adenocarcinomas of the lung. Cancer Res 66: 7466–7472. 16885343 10.1158/0008-5472.CAN-06-1191

[18] Hess KR, Anderson K, Symmans WF, Valero V, Ibrahim N, et al (2006) Pharmacogenomic predictor of sensitivity to preoperative chemotherapy with paclitaxel and fluorouracil, doxorubicin, and cyclophosphamide in breast cancer. J Clin Oncol 24: 4236–4244. 16896004 10.1200/JCO.2006.05.6861

[19] Hwang WL, Yang MH, Tsai ML, Lan HY, Su SH, et al (2011) SNAIL regulates interleukin-8 expression, stem cell-like activity, and tumorigenicity of human colorectal carcinoma cells. Gastroenterology 141: 279–291, 291 e271–275. 21640118 10.1053/j.gastro.2011.04.008

[20] Micalizzi DS, Christensen KL, Jedlicka P, Coletta RD, Baron AE, et al (2009) The Six1 homeoprotein induces human mammary carcinoma cells to undergo epithelial-mesenchymal transition and metastasis in mice through increasing TGF-beta signaling. J Clin Invest 119: 2678–2690. 19726885 10.1172/JCI37815 PMC2735914

[21] Baniwal SK, Khalid O, Gabet Y, Shah RR, Purcell DJ, et al (2010) Runx2 transcriptome of prostate cancer cells: insights into invasiveness and bone metastasis. Mol Cancer 9: 258. 20863401 10.1186/1476-4598-9-258 PMC2955618

[22] Micalizzi DS, Wang CA, Farabaugh SM, Schiemann WP, Ford HL (2010) Homeoprotein Six1 increases TGF-beta type I receptor and converts TGF-beta signaling from suppressive to supportive for tumor growth. Cancer Res 70: 10371–10380. 21056993 10.1158/0008-5472.CAN-10-1354 PMC3072046

[23] Farabaugh SM, Micalizzi DS, Jedlicka P, Zhao R, Ford HL (2012) Eya2 is required to mediate the pro-metastatic functions of Six1 via the induction of TGF-beta signaling, epithelial-mesenchymal transition, and cancer stem cell properties. Oncogene 31: 552–562. 21706047 10.1038/onc.2011.259 PMC3183358

[24] Lee KS, Hong SH, Bae SC (2002) Both the Smad and p38 MAPK pathways play a crucial role in Runx2 expression following induction by transforming growth factor-beta and bone morphogenetic protein. Oncogene 21: 7156–7163. 12370805 10.1038/sj.onc.1205937

[25] Chimge NO, Baniwal SK, Little GH, Chen YB, Kahn M, et al (2011) Regulation of breast cancer metastasis by Runx2 and estrogen signaling: the role of SNAI2. Breast Cancer Res 13: R127. 22151997 10.1186/bcr3073 PMC3326569

[26] van Zijl F, Mall S, Machat G, Pirker C, Zeillinger R, et al (2011) A human model of epithelial to mesenchymal transition to monitor drug efficacy in hepatocellular carcinoma progression. Mol Cancer Ther 10: 850–860. 21364009 10.1158/1535-7163.MCT-10-0917

[27] Ip YT, Park RE, Kosman D, Yazdanbakhsh K, Levine M (1992) dorsal-twist interactions establish snail expression in the presumptive mesoderm of the Drosophila embryo. Genes Dev 6: 1518–1530. 1644293 10.1101/gad.6.8.1518

[28] Ke XS, Li WC, Hovland R, Qu Y, Liu RH, et al (2011) Reprogramming of cell junction modules during stepwise epithelial to mesenchymal transition and accumulation of malignant features in vitro in a prostate cell model. Exp Cell Res 317: 234–247. 20969863 10.1016/j.yexcr.2010.10.009

[29] Ohashi S, Natsuizaka M, Naganuma S, Kagawa S, Kimura S, et al (2011) A NOTCH3-mediated squamous cell differentiation program limits expansion of EMT-competent cells that express the ZEB transcription factors. Cancer Res 71: 6836–6847. 21890822 10.1158/0008-5472.CAN-11-0846 PMC3206139

[30] Zoltan-Jones A, Huang L, Ghatak S, Toole BP (2003) Elevated hyaluronan production induces mesenchymal and transformed properties in epithelial cells. J Biol Chem 278: 45801–45810. 12954618 10.1074/jbc.M308168200

[31] Ren D, Minami Y, Nishita M (2011) Critical role of Wnt5a-Ror2 signaling in motility and invasiveness of carcinoma cells following Snail-mediated epithelial-mesenchymal transition. Genes Cells 16: 304–315. 21342370 10.1111/j.1365-2443.2011.01487.x

[32] Kumar S, Park SH, Cieply B, Schupp J, Killiam E, et al (2011) A pathway for the control of anoikis sensitivity by E-cadherin and epithelial-to-mesenchymal transition. Mol Cell Biol 31: 4036–4051. 21746881 10.1128/MCB.01342-10 PMC3187352

[33] Chen LM, Verity NJ, Chai KX (2009) Loss of prostasin (PRSS8) in human bladder transitional cell carcinoma cell lines is associated with epithelial-mesenchymal transition (EMT). BMC Cancer 9: 377. 19849847 10.1186/1471-2407-9-377 PMC2770574

[34] Sung YM, Xu X, Sun J, Mueller D, Sentissi K, et al (2009) Tumor suppressor function of Syk in human MCF10A in vitro and normal mouse mammary epithelium in vivo. PLoS One 4: e7445. 19829710 10.1371/journal.pone.0007445 PMC2759536

[35] Talieri M, Alexopoulou DK, Scorilas A, Kypraios D, Arnogiannaki N, et al (2011) Expression analysis and clinical evaluation of kallikrein-related peptidase 10 (KLK10) in colorectal cancer. Tumour Biol 32: 737–744. 21487810 10.1007/s13277-011-0175-4

[36] Seibold S, Rudroff C, Weber M, Galle J, Wanner C, et al (2003) Identification of a new tumor suppressor gene located at chromosome 8p21.3-22. FASEB J 17: 1180–1182. 12692079 10.1096/fj.02-0934fje

[37] Mandal S, Abebe F, Chaudhary J (2011) 2′-5′ oligoadenylate synthetase 1 polymorphism is associated with prostate cancer. Cancer 117: 5509–5518. 21638280 10.1002/cncr.26219 PMC3167978

[38] Chou RH, Wen HC, Liang WG, Lin SC, Yuan HW, et al (2012) Suppression of the invasion and migration of cancer cells by SERPINB family genes and their derived peptides. Oncol Rep 27: 238–245. 21993616 10.3892/or.2011.1497

[39] Choi YL, Bocanegra M, Kwon MJ, Shin YK, Nam SJ, et al (2010) LYN is a mediator of epithelial-mesenchymal transition and a target of dasatinib in breast cancer. Cancer Res 70: 2296–2306. 20215510 10.1158/0008-5472.CAN-09-3141 PMC2869247

[40] Yamamoto H, Okumura K, Toshima S, Mukaisho K, Sugihara H, et al (2009) FXYD3 protein involved in tumor cell proliferation is overproduced in human breast cancer tissues. Biol Pharm Bull 32: 1148–1154. 19571376 10.1248/bpb.32.1148

[41] Yamamoto H, Mukaisho K, Sugihara H, Hattori T, Asano S (2011) Down-regulation of FXYD3 is induced by transforming growth factor-beta signaling via ZEB1/deltaEF1 in human mammary epithelial cells. Biol Pharm Bull 34: 324–329. 21372379 10.1248/bpb.34.324

[42] Diamandis EP, Goodglick L, Planque C, Thornquist MD (2011) Pentraxin-3 is a novel biomarker of lung carcinoma. Clin Cancer Res 17: 2395–2399. 21257721 10.1158/1078-0432.CCR-10-3024

[43] Rouleau C, Roy A, St Martin T, Dufault MR, Boutin P, et al (2006) Protein tyrosine phosphatase PRL-3 in malignant cells and endothelial cells: expression and function. Mol Cancer Ther 5: 219–229. 16505094 10.1158/1535-7163.MCT-05-0289

[44] Kuk C, Gunawardana CG, Soosaipillai A, Kobayashi H, Li L, et al (2010) Nidogen-2: a new serum biomarker for ovarian cancer. Clin Biochem 43: 355–361. 19883638 10.1016/j.clinbiochem.2009.10.012 PMC3109863

[45] Marr HS, Edgell CJ (2003) Testican-1 inhibits attachment of Neuro-2a cells. Matrix Biol 22: 259–266. 12853036 10.1016/s0945-053x(03)00036-2

[46] Nakada M, Yamada A, Takino T, Miyamori H, Takahashi T, et al (2001) Suppression of membrane-type 1 matrix metalloproteinase (MMP)-mediated MMP-2 activation and tumor invasion by testican 3 and its splicing variant gene product, N-Tes. Cancer Res 61: 8896–8902. 11751414

[47] Junnila S, Kokkola A, Mizuguchi T, Hirata K, Karjalainen-Lindsberg ML, et al (2010) Gene expression analysis identifies over-expression of CXCL1, SPARC, SPP1, and SULF1 in gastric cancer. Genes Chromosomes Cancer 49: 28–39. 19780053 10.1002/gcc.20715

[48] Yue X, Li X, Nguyen HT, Chin DR, Sullivan DE, et al (2008) Transforming growth factor-beta1 induces heparan sulfate 6-O-endosulfatase 1 expression in vitro and in vivo. J Biol Chem 283: 20397–20407. 18503048 10.1074/jbc.M802850200 PMC2459296

[49] Sun W, Wei X, Kesavan K, Garrington TP, Fan R, et al (2003) MEK kinase 2 and the adaptor protein Lad regulate extracellular signal-regulated kinase 5 activation by epidermal growth factor via Src. Mol Cell Biol 23: 2298–2308. 12640115 10.1128/MCB.23.7.2298-2308.2003 PMC150715

[50] Wen J, Nikitakis NG, Chaisuparat R, Greenwell-Wild T, Gliozzi M, et al (2011) Secretory leukocyte protease inhibitor (SLPI) expression and tumor invasion in oral squamous cell carcinoma. Am J Pathol 178: 2866–2878. 21641406 10.1016/j.ajpath.2011.02.017 PMC3124294

[51] Amiano NO, Costa MJ, Reiteri RM, Payes C, Guerrieri D, et al (2012) Antitumor effect of SLPI on mammary but not colon tumor growth. J Cell Physiol 10.1002/jcp.24153 22767220

[52] Choi BD, Jeong SJ, Wang G, Park JJ, Lim DS, et al (2011) Secretory leukocyte protease inhibitor is associated with MMP-2 and MMP-9 to promote migration and invasion in SNU638 gastric cancer cells. Int J Mol Med 28: 527–534. 21687932 10.3892/ijmm.2011.726

[53] Gentleman RC, Carey VJ, Bates DM, Bolstad B, Dettling M, et al (2004) Bioconductor: open software development for computational biology and bioinformatics. Genome Biol 5: R80. 15461798 10.1186/gb-2004-5-10-r80 PMC545600

[54] R Development Core Team (2011) R: A language and environment for statistical computing. Vienna, Austria: R Foundation for Statistical Computing.

[55] Warnes GR, Bolker B, Gorjanc G, Grothendieck G, Korosec A, et al. (2011) gdata: Various R programming tools for data manipulation. R package version 2.8.2. CRAN website. Available: http://CRAN.R-project.org/package=gdata. Accessed 2012 June 25.

[56] Warnes GR, Bolker B, Bonebakker L, Gentleman R, Huber W, et al. (2011) gplots: Various R programming tools for plotting data. R package version 2.10.1. CRAN website. Available: http://CRAN.R-project.org/package=gplots. Accessed 2012 June 25.

[57] Day A (2007) heatmap.plus: Heatmap with more sensible behavior. R package version 1.3. CRAN website. Available: http://CRAN.R-project.org/package=heatmap.plus. Accessed 2012 June 25.

[58] Medina I, Carbonell J, Pulido L, Madeira SC, Goetz S, et al (2010) Babelomics: an integrative platform for the analysis of transcriptomics, proteomics and genomic data with advanced functional profiling. Nucleic Acids Res 38: W210–213. 20478823 10.1093/nar/gkq388 PMC2896184

[59] Zhang B, Kirov S, Snoddy J (2005) WebGestalt: an integrated system for exploring gene sets in various biological contexts. Nucleic Acids Res 33: W741–748. 15980575 10.1093/nar/gki475 PMC1160236

[60] Thernau T (2012) A Package for Survival Analysis in S. CRAN website. Available: http://CRAN.R-project.org/package=survival. Accessed 2012 June 25.

[61] Andarawewa KL, Erickson AC, Chou WS, Costes SV, Gascard P, et al (2007) Ionizing radiation predisposes nonmalignant human mammary epithelial cells to undergo transforming growth factor beta induced epithelial to mesenchymal transition. Cancer Res 67: 8662–8670. 17875706 10.1158/0008-5472.CAN-07-1294

[62] Tay PN, Tan P, Lan Y, Leung CH, Laban M, et al (2010) Palladin, an actin-associated protein, is required for adherens junction formation and intercellular adhesion in HCT116 colorectal cancer cells. Int J Oncol 37: 909–926. 20811713 10.3892/ijo_00000742

[63] Drake JM, Strohbehn G, Bair TB, Moreland JG, Henry MD (2009) ZEB1 enhances transendothelial migration and represses the epithelial phenotype of prostate cancer cells. Mol Biol Cell 20: 2207–2217. 19225155 10.1091/mbc.E08-10-1076 PMC2669028

[64] Sartor MA, Mahavisno V, Keshamouni VG, Cavalcoli J, Wright Z, et al (2010) ConceptGen: a gene set enrichment and gene set relation mapping tool. Bioinformatics 26: 456–463. 20007254 10.1093/bioinformatics/btp683 PMC2852214

[65] Papageorgis P, Lambert AW, Ozturk S, Gao F, Pan H, et al (2010) Smad signaling is required to maintain epigenetic silencing during breast cancer progression. Cancer Res 70: 968–978. 20086175 10.1158/0008-5472.CAN-09-1872 PMC2946209

[66] Hills CE, Willars GB, Brunskill NJ (2010) Proinsulin C-peptide antagonizes the profibrotic effects of TGF-beta1 via up-regulation of retinoic acid and HGF-related signaling pathways. Mol Endocrinol 24: 822–831. 20197308 10.1210/me.2009-0391 PMC5417534

[67] Leshem O, Madar S, Kogan-Sakin I, Kamer I, Goldstein I, et al (2011) TMPRSS2/ERG promotes epithelial to mesenchymal transition through the ZEB1/ZEB2 axis in a prostate cancer model. PLoS One 6: e21650. 21747944 10.1371/journal.pone.0021650 PMC3128608

[68] Maupin KA, Sinha A, Eugster E, Miller J, Ross J, et al (2010) Glycogene expression alterations associated with pancreatic cancer epithelial-mesenchymal transition in complementary model systems. PLoS One 5: e13002. 20885998 10.1371/journal.pone.0013002 PMC2946336

[69] Hesling C, Fattet L, Teyre G, Jury D, Gonzalo P, et al (2011) Antagonistic regulation of EMT by TIF1gamma and Smad4 in mammary epithelial cells. EMBO Rep 12: 665–672. 21597466 10.1038/embor.2011.78 PMC3128966

[70] Wang L, Mezencev R, Bowen NJ, Matyunina LV, McDonald JF (2011) Isolation and characterization of stem-like cells from a human ovarian cancer cell line. Mol Cell Biochem 363: 257–268. 22160925 10.1007/s11010-011-1178-6

