# Supplementary material for: Meta-Analysis of Gene Expression Signatures Defining the Epithelial to Mesenchymal Transition during Cancer Progression
Source: PLoS One. 2012 Dec 10;7(12):e51136. doi: 10.1371/journal.pone.0051136 (PMC3519484; doi:10.1371/journal.pone.0051136)
Supplement: Figure S1 — Cluster analysis of genes shared between at least 14 GES datasets shows persistent and distinct clusters. (PDF) [file pone.0051136.s001.pdf]

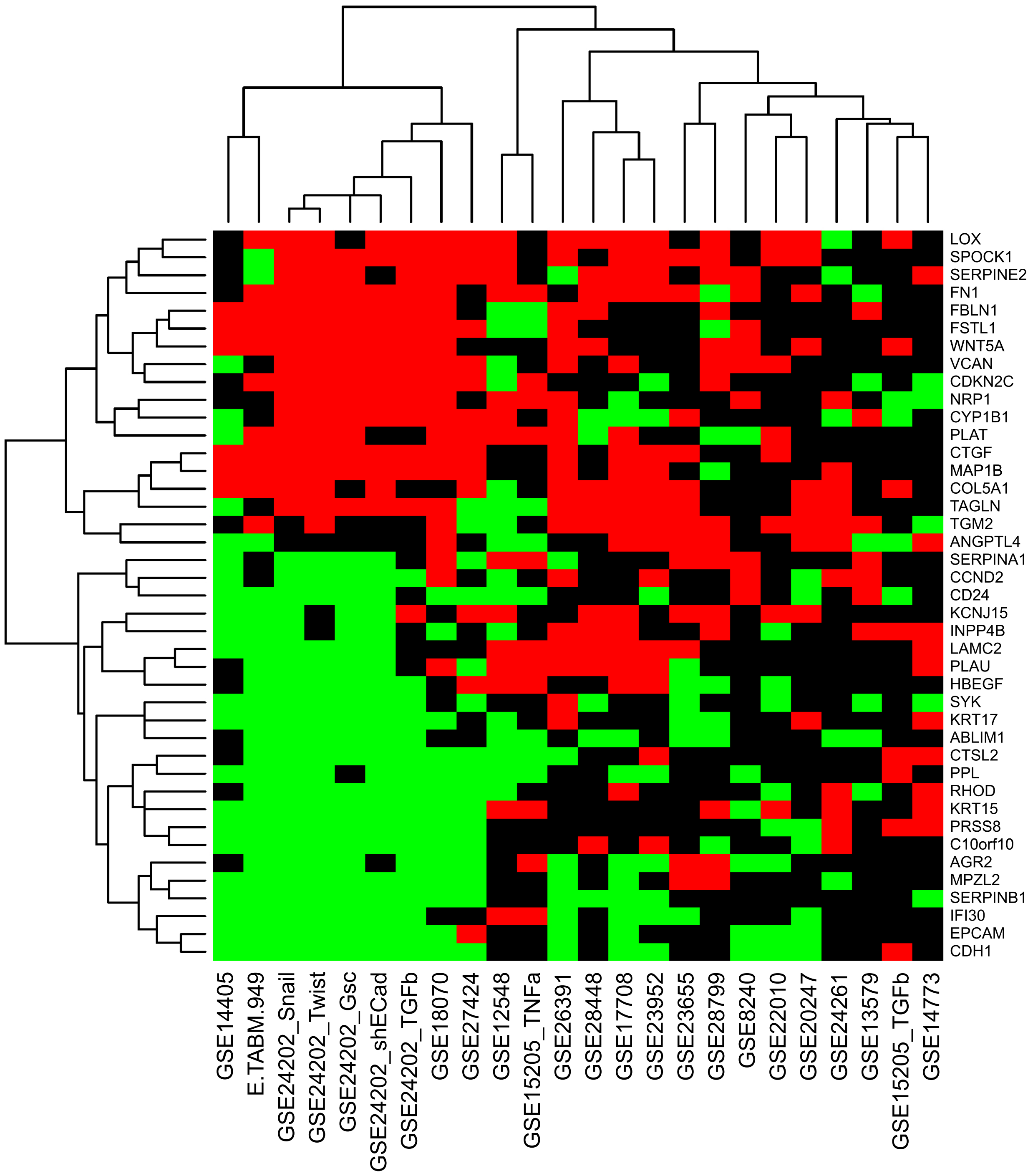

Figure S1: Cluster analysis of genes shared between at least 14 GES datasets shows persistent and distinct clusters. Genes shared between at least 14 out of 24 datasets were used for Manhattan hierarchical clustering. The type of regulation within a particular study was visualized via heatmap. Columns: genes shared between at least 14 GES datasets (n=41); rows: analyzed GES (24 datasets in total); green: downregulated genes; red: upregulated genes; black: genes not regulated. GSE: Gene expression omnibus (GEO) series record; E.TABM: ArrayExpress (AE) series record; TGF, transforming growth factor; TNF, tumor necrosis factor.
