# Supplementary material for: Meta-Analysis of Gene Expression Signatures Defining the Epithelial to Mesenchymal Transition during Cancer Progression
Source: PLoS One. 2012 Dec 10;7(12):e51136. doi: 10.1371/journal.pone.0051136 (PMC3519484; doi:10.1371/journal.pone.0051136)
Supplement: Figure S2 — The 130 genes EMT-core list and the 365 genes list exhibit comparable enrichment ratios of GO molecular functions. (PDF) [file pone.0051136.s002.pdf]

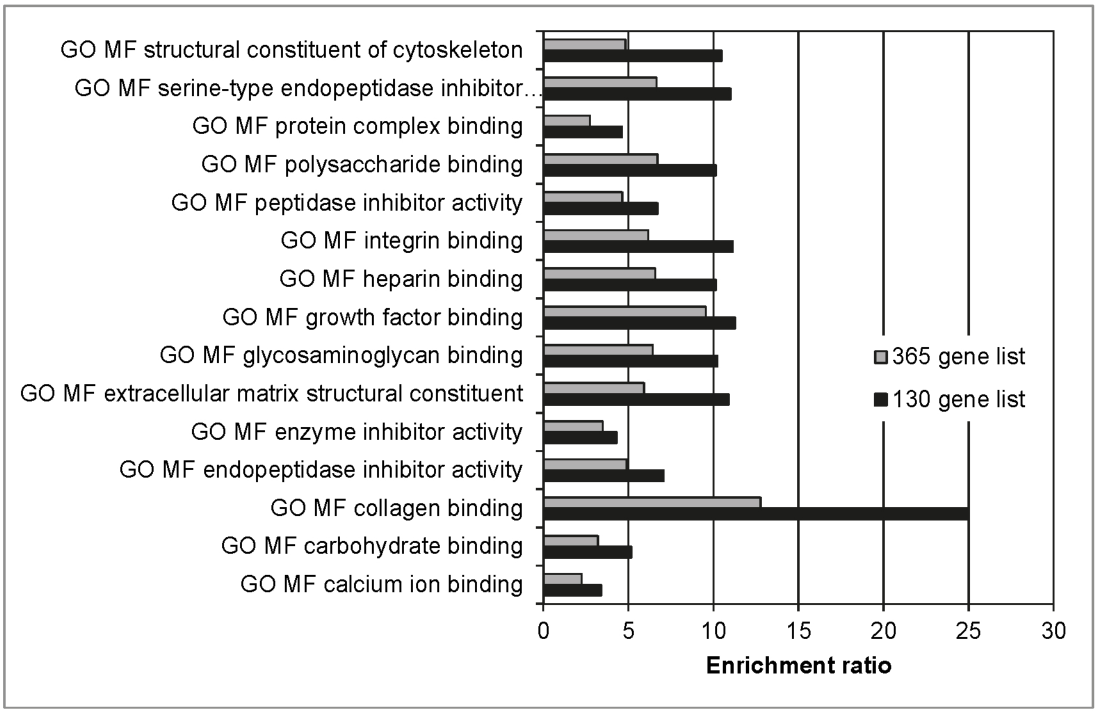

Figure S2: The 130 genes EMT-core list and the 365 genes list exhibit comparable enrichment ratios of GO molecular functions. The enrichment ratio is the number of observed genes divided by the number of expected genes for a given term or pathway. Enrichment ratios were obtained from WebGestalt or calculated with data from FatiGO. GO, gene ontology; BP, biological process; MF, molecular function; KEGG, Kyoto encyclopedia of genes and genomes.
