# Supplementary material for: Meta-Analysis of Gene Expression Signatures Defining the Epithelial to Mesenchymal Transition during Cancer Progression
Source: PLoS One. 2012 Dec 10;7(12):e51136. doi: 10.1371/journal.pone.0051136 (PMC3519484; doi:10.1371/journal.pone.0051136)
Supplement: Figure S3 — Flow chart depicting the generation of the EMT-core gene list. (PDF) [file pone.0051136.s003.pdf]

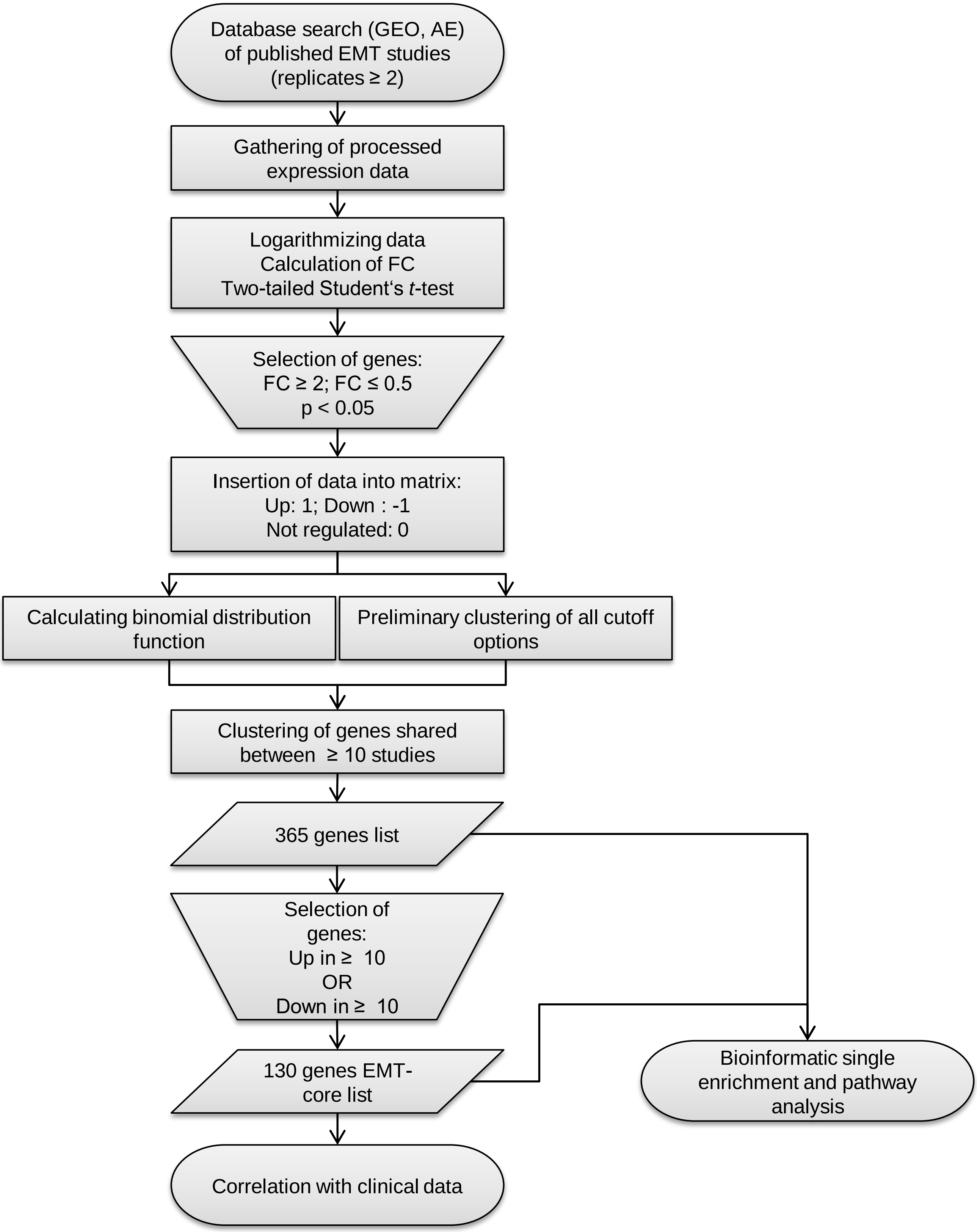


**Figure S3: Flow chart depicting the generation of the EMT-core gene list**. Overview of the meta-analysis, the generation of the 365 genes list, the EMT-core list as well as the single enrichment analysis and the correlation with clinical data. GEO, Gene expression Omnibus; AE, Array Express; EMT, epithelial to mesenchymal transition; FC, Fold change; Up, upregulated; Down, downregulated.
