# Supplementary material for: Meta-Analysis of Gene Expression Signatures Defining the Epithelial to Mesenchymal Transition during Cancer Progression
Source: PLoS One. 2012 Dec 10;7(12):e51136. doi: 10.1371/journal.pone.0051136 (PMC3519484; doi:10.1371/journal.pone.0051136)
Supplement: Table S2 — List of 365 genes significantly regulated in at least 10 GES datasets. (DOC) [file pone.0051136.s005.doc]

**Table S2:** List of 365 genes significantly regulated in at least 10 GES datasets.

| **Gene Symbol** | **EntrezID** | **Gene Name** |
| --- | --- | --- |
| ABCA1 | 19 | ATP-binding cassette, sub-family A (ABC1), member 1 |
| ABCA12 | 26154 | ATP-binding cassette, sub-family A (ABC1), member 12 |
| ABCC3 | 8714 | ATP-binding cassette, sub-family C (CFTR/MRP), member 3 |
| ABLIM1 | 3983 | actin binding LIM protein 1 |
| ACTA2 | 59 | actin, alpha 2, smooth muscle, aorta |
| ADAM12 | 8038 | ADAM metallopeptidase domain 12 |
| ADM | 133 | adrenomedullin |
| ADRB2 | 154 | adrenergic, beta-2-, receptor, surface |
| AGR2 | 10551 | anterior gradient homolog 2 (Xenopus laevis) |
| AK4 | 205 | adenylate kinase 4 |
| AKAP12 | 9590 | A kinase (PRKA) anchor protein 12 |
| AKR1C1 | 1645 | aldo-keto reductase family 1, member C1 (dihydrodiol dehydrogenase 1; 20-alpha (3-alpha)-hydroxysteroid dehydrogenase) |
| AKR1C2 | 1646 | aldo-keto reductase family 1, member C2 (dihydrodiol dehydrogenase 2; bile acid binding protein; 3-alpha hydroxysteroid dehydrogenase, type III) |
| AKR1C3 | 8644 | aldo-keto reductase family 1, member C3 (3-alpha hydroxysteroid dehydrogenase, type II) |
| ALDH1A3 | 220 | aldehyde dehydrogenase 1 family, member A3 |
| ANGPTL4 | 51129 | angiopoietin-like 4 |
| ANK3 | 288 | ankyrin 3, node of Ranvier (ankyrin G) |
| ANXA6 | 309 | annexin A6 |
| APOBEC3B | 9582 | apolipoprotein B mRNA editing enzyme, catalytic polypeptide-like 3B |
| AREG | 374 | amphiregulin |
| ARHGAP26 | 23092 | Rho GTPase activating protein 26 |
| ARID5B | 84159 | AT rich interactive domain 5B (MRF1-like) |
| BAMBI | 25805 | BMP and activin membrane-bound inhibitor homolog (Xenopus laevis) |
| BDKRB2 | 624 | bradykinin receptor B2 |
| BGN | 633 | biglycan |
| BIK | 638 | BCL2-interacting killer (apoptosis-inducing) |
| BIN1 | 274 | bridging integrator 1 |
| BIRC3 | 330 | baculoviral IAP repeat containing 3 |
| C10orf10 | 11067 | chromosome 10 open reading frame 10 |
| C1R | 715 | complement component 1, r subcomponent |
| C5orf13 | 9315 | chromosome 5 open reading frame 13 |
| C9orf3 | 84909 | chromosome 9 open reading frame 3 |
| CA2 | 760 | carbonic anhydrase II |
| CALD1 | 800 | caldesmon 1 |
| CASP1 | 834 | caspase 1, apoptosis-related cysteine peptidase (interleukin 1, beta, convertase) |
| CBR3 | 874 | carbonyl reductase 3 |
| CCL20 | 6364 | chemokine (C-C motif) ligand 20 |
| CCND2 | 894 | cyclin D2 |
| CCNE2 | 9134 | cyclin E2 |
| CD24 | 100133941 | CD24 molecule |
| CD44 | 960 | CD44 molecule (Indian blood group) |
| CDH1 | 999 | cadherin 1, type 1, E-cadherin (epithelial) |
| CDH11 | 1009 | cadherin 11, type 2, OB-cadherin (osteoblast) |
| CDH2 | 1000 | cadherin 2, type 1, N-cadherin (neuronal) |
| CDH3 | 1001 | cadherin 3, type 1, P-cadherin (placental) |
| CDK14 | 5218 | cyclin-dependent kinase 14 |
| CDKN2C | 1031 | cyclin-dependent kinase inhibitor 2C (p18, inhibits CDK4) |
| CDS1 | 1040 | CDP-diacylglycerol synthase (phosphatidate cytidylyltransferase) 1 |
| CHST2 | 9435 | carbohydrate (N-acetylglucosamine-6-O) sulfotransferase 2 |
| CITED2 | 10370 | Cbp/p300-interacting transactivator, with Glu/Asp-rich carboxy-terminal domain, 2 |
| CNTN1 | 1272 | contactin 1 |
| COBLL1 | 22837 | COBL-like 1 |
| COL1A1 | 1277 | collagen, type I, alpha 1 |
| COL3A1 | 1281 | collagen, type III, alpha 1 |
| COL4A1 | 1282 | collagen, type IV, alpha 1 |
| COL5A1 | 1289 | collagen, type V, alpha 1 |
| COL6A1 | 1291 | collagen, type VI, alpha 1 |
| COL6A3 | 1293 | collagen, type VI, alpha 3 |
| CPM | 1368 | carboxypeptidase M |
| CRABP2 | 1382 | cellular retinoic acid binding protein 2 |
| CREB3L1 | 90993 | cAMP responsive element binding protein 3-like 1 |
| CST6 | 1474 | cystatin E/M |
| CTGF | 1490 | connective tissue growth factor |
| CTH | 1491 | cystathionase (cystathionine gamma-lyase) |
| CTSL2 | 1515 | cathepsin L2 |
| CXADR | 1525 | coxsackie virus and adenovirus receptor |
| CXCL1 | 2919 | chemokine (C-X-C motif) ligand 1 (melanoma growth stimulating activity, alpha) |
| CXCL16 | 58191 | chemokine (C-X-C motif) ligand 16 |
| CXCL2 | 2920 | chemokine (C-X-C motif) ligand 2 |
| CYP1B1 | 1545 | cytochrome P450, family 1, subfamily B, polypeptide 1 |
| CYP4F11 | 57834 | cytochrome P450, family 4, subfamily F, polypeptide 11 |
| DAB2 | 1601 | disabled homolog 2, mitogen-responsive phosphoprotein (Drosophila) |
| DAPK1 | 1612 | death-associated protein kinase 1 |
| DCN | 1634 | decorin |
| DDR1 | 780 | discoidin domain receptor tyrosine kinase 1 |
| DHRS2 | 10202 | dehydrogenase/reductase (SDR family) member 2 |
| DLC1 | 10395 | deleted in liver cancer 1 |
| DNAJB4 | 11080 | DnaJ (Hsp40) homolog, subfamily B, member 4 |
| DPYD | 1806 | dihydropyrimidine dehydrogenase |
| DPYSL3 | 1809 | dihydropyrimidinase-like 3 |
| DSC2 | 1824 | desmocollin 2 |
| DSG3 | 1830 | desmoglein 3 |
| DUSP1 | 1843 | dual specificity phosphatase 1 |
| DUSP10 | 11221 | dual specificity phosphatase 10 |
| DUSP6 | 1848 | dual specificity phosphatase 6 |
| ELF3 | 1999 | E74-like factor 3 (ets domain transcription factor, epithelial-specific ) |
| ELL2 | 22936 | elongation factor, RNA polymerase II, 2 |
| EML1 | 2009 | echinoderm microtubule associated protein like 1 |
| EMP3 | 2014 | epithelial membrane protein 3 |
| ENAH | 55740 | enabled homolog (Drosophila) |
| ENPP1 | 5167 | ectonucleotide pyrophosphatase/phosphodiesterase 1 |
| ENPP2 | 5168 | ectonucleotide pyrophosphatase/phosphodiesterase 2 |
| EPCAM | 4072 | epithelial cell adhesion molecule |
| EPHA1 | 2041 | EPH receptor A1 |
| EPHA4 | 2043 | EPH receptor A4 |
| EPHB2 | 2048 | EPH receptor B2 |
| EPHX1 | 2052 | epoxide hydrolase 1, microsomal (xenobiotic) |
| EREG | 2069 | epiregulin |
| ETV1 | 2115 | ets variant 1 |
| EXPH5 | 23086 | exophilin 5 |
| F2R | 2149 | coagulation factor II (thrombin) receptor |
| F3 | 2152 | coagulation factor III (thromboplastin, tissue factor) |
| FADS1 | 3992 | fatty acid desaturase 1 |
| FAM169A | 26049 | family with sequence similarity 169, member A |
| FBLN1 | 2192 | fibulin 1 |
| FBLN5 | 10516 | fibulin 5 |
| FBN1 | 2200 | fibrillin 1 |
| FERMT1 | 55612 | fermitin family member 1 |
| FGF2 | 2247 | fibroblast growth factor 2 (basic) |
| FGFBP1 | 9982 | fibroblast growth factor binding protein 1 |
| FGFR1 | 2260 | fibroblast growth factor receptor 1 |
| FGFR2 | 2263 | fibroblast growth factor receptor 2 |
| FGFR3 | 2261 | fibroblast growth factor receptor 3 |
| FILIP1L | 11259 | filamin A interacting protein 1-like |
| FLOT1 | 10211 | flotillin 1 |
| FLRT2 | 23768 | fibronectin leucine rich transmembrane protein 2 |
| FN1 | 2335 | fibronectin 1 |
| FRMD4A | 55691 | FERM domain containing 4A |
| FST | 10468 | follistatin |
| FSTL1 | 11167 | follistatin-like 1 |
| FXYD3 | 5349 | FXYD domain containing ion transport regulator 3 |
| FZD7 | 8324 | frizzled homolog 7 (Drosophila) |
| GADD45B | 4616 | growth arrest and DNA-damage-inducible, beta |
| GALNT10 | 55568 | UDP-N-acetyl-alpha-D-galactosamine:polypeptide N-acetylgalactosaminyltransferase 10 (GalNAc-T10) |
| GCLC | 2729 | glutamate-cysteine ligase, catalytic subunit |
| GEM | 2669 | GTP binding protein overexpressed in skeletal muscle |
| GJB3 | 2707 | gap junction protein, beta 3, 31kDa |
| GLIPR1 | 11010 | GLI pathogenesis-related 1 |
| GLRX | 2745 | glutaredoxin (thioltransferase) |
| GLS | 2744 | glutaminase |
| GNAL | 2774 | guanine nucleotide binding protein (G protein), alpha activating activity polypeptide, olfactory type |
| GPX3 | 2878 | glutathione peroxidase 3 (plasma) |
| GULP1 | 51454 | GULP, engulfment adaptor PTB domain containing 1 |
| HAS2 | 3037 | hyaluronan synthase 2 |
| HBEGF | 1839 | heparin-binding EGF-like growth factor |
| HDAC9 | 9734 | histone deacetylase 9 |
| HMGA2 | 8091 | high mobility group AT-hook 2 |
| HMOX1 | 3162 | heme oxygenase (decycling) 1 |
| HPGD | 3248 | hydroxyprostaglandin dehydrogenase 15-(NAD) |
| HS3ST1 | 9957 | heparan sulfate (glucosamine) 3-O-sulfotransferase 1 |
| HS3ST3A1 | 9955 | heparan sulfate (glucosamine) 3-O-sulfotransferase 3A1 |
| HSD17B2 | 3294 | hydroxysteroid (17-beta) dehydrogenase 2 |
| HTRA1 | 5654 | HtrA serine peptidase 1 |
| ID2 | 3398 | inhibitor of DNA binding 2, dominant negative helix-loop-helix protein |
| IFI30 | 10437 | interferon, gamma-inducible protein 30 |
| IFI44 | 10561 | interferon-induced protein 44 |
| IFI44L | 10964 | interferon-induced protein 44-like |
| IFI6 | 2537 | interferon, alpha-inducible protein 6 |
| IFIT1 | 3434 | interferon-induced protein with tetratricopeptide repeats 1 |
| IFITM1 | 8519 | interferon induced transmembrane protein 1 (9-27) |
| IGFBP2 | 3485 | insulin-like growth factor binding protein 2, 36kDa |
| IGFBP3 | 3486 | insulin-like growth factor binding protein 3 |
| IGFBP4 | 3487 | insulin-like growth factor binding protein 4 |
| IGFBP5 | 3488 | insulin-like growth factor binding protein 5 |
| IGFBP7 | 3490 | insulin-like growth factor binding protein 7 |
| IL18 | 3606 | interleukin 18 (interferon-gamma-inducing factor) |
| IL1A | 3552 | interleukin 1, alpha |
| IL1B | 3553 | interleukin 1, beta |
| IL1R1 | 3554 | interleukin 1 receptor, type I |
| IL1R2 | 7850 | interleukin 1 receptor, type II |
| IL1RAP | 3556 | interleukin 1 receptor accessory protein |
| IL1RN | 3557 | interleukin 1 receptor antagonist |
| IL32 | 9235 | interleukin 32 |
| IL4R | 3566 | interleukin 4 receptor |
| IL7R | 3575 | interleukin 7 receptor |
| IL8 | 3576 | interleukin 8 |
| INHBA | 3624 | inhibin, beta A |
| INPP4B | 8821 | inositol polyphosphate-4-phosphatase, type II, 105kDa |
| INSIG1 | 3638 | insulin induced gene 1 |
| IQGAP2 | 10788 | IQ motif containing GTPase activating protein 2 |
| ITGA2 | 3673 | integrin, alpha 2 (CD49B, alpha 2 subunit of VLA-2 receptor) |
| ITGB4 | 3691 | integrin, beta 4 |
| ITGB5 | 3693 | integrin, beta 5 |
| JAG2 | 3714 | jagged 2 |
| JUP | 3728 | junction plakoglobin |
| KCNJ15 | 3772 | potassium inwardly-rectifying channel, subfamily J, member 15 |
| KCNK1 | 3775 | potassium channel, subfamily K, member 1 |
| KLK10 | 5655 | kallikrein-related peptidase 10 |
| KLK7 | 5650 | kallikrein-related peptidase 7 |
| KRT15 | 3866 | keratin 15 |
| KRT16 | 3868 | keratin 16 |
| KRT17 | 3872 | keratin 17 |
| KRT18 | 3875 | keratin 18 |
| KRT6A | 3853 | keratin 6A |
| KRT81 | 3887 | keratin 81 |
| LAD1 | 3898 | ladinin 1 |
| LAMA3 | 3909 | laminin, alpha 3 |
| LAMA4 | 3910 | laminin, alpha 4 |
| LAMB3 | 3914 | laminin, beta 3 |
| LAMC2 | 3918 | laminin, gamma 2 |
| LCN2 | 3934 | lipocalin 2 |
| LIMK2 | 3985 | LIM domain kinase 2 |
| LOX | 4015 | lysyl oxidase |
| LRP8 | 7804 | low density lipoprotein receptor-related protein 8, apolipoprotein e receptor |
| LSR | 51599 | lipolysis stimulated lipoprotein receptor |
| LSS | 4047 | lanosterol synthase (2,3-oxidosqualene-lanosterol cyclase) |
| LTBP1 | 4052 | latent transforming growth factor beta binding protein 1 |
| LTBP2 | 4053 | latent transforming growth factor beta binding protein 2 |
| LUM | 4060 | lumican |
| MAF | 4094 | v-maf musculoaponeurotic fibrosarcoma oncogene homolog (avian) |
| MALL | 7851 | mal, T-cell differentiation protein-like |
| MAN1A1 | 4121 | mannosidase, alpha, class 1A, member 1 |
| MAP1B | 4131 | microtubule-associated protein 1B |
| MAP7 | 9053 | microtubule-associated protein 7 |
| MARCKS | 4082 | myristoylated alanine-rich protein kinase C substrate |
| MBP | 4155 | myelin basic protein |
| METTL7A | 25840 | methyltransferase like 7A |
| MFAP2 | 4237 | microfibrillar-associated protein 2 |
| MGLL | 11343 | monoglyceride lipase |
| MICAL2 | 9645 | microtubule associated monoxygenase, calponin and LIM domain containing 2 |
| MME | 4311 | membrane metallo-endopeptidase |
| MMP1 | 4312 | matrix metallopeptidase 1 (interstitial collagenase) |
| MMP19 | 4327 | matrix metallopeptidase 19 |
| MMP2 | 4313 | matrix metallopeptidase 2 (gelatinase A, 72kDa gelatinase, 72kDa type IV collagenase) |
| MMP9 | 4318 | matrix metallopeptidase 9 (gelatinase B, 92kDa gelatinase, 92kDa type IV collagenase) |
| MN1 | 4330 | meningioma (disrupted in balanced translocation) 1 |
| MPZL2 | 10205 | myelin protein zero-like 2 |
| MST1R | 4486 | macrophage stimulating 1 receptor (c-met-related tyrosine kinase) |
| MTSS1 | 9788 | metastasis suppressor 1 |
| MTUS1 | 57509 | microtubule associated tumor suppressor 1 |
| MYL9 | 10398 | myosin, light chain 9, regulatory |
| MYLK | 4638 | myosin light chain kinase |
| MYO10 | 4651 | myosin X |
| NAP1L3 | 4675 | nucleosome assembly protein 1-like 3 |
| NAV3 | 89795 | neuron navigator 3 |
| NCF2 | 4688 | neutrophil cytosolic factor 2 |
| NDRG1 | 10397 | N-myc downstream regulated 1 |
| NEBL | 10529 | nebulette |
| NEDD9 | 4739 | neural precursor cell expressed, developmentally down-regulated 9 |
| NFE2L3 | 9603 | nuclear factor (erythroid-derived 2)-like 3 |
| NID2 | 22795 | nidogen 2 (osteonidogen) |
| NMU | 10874 | neuromedin U |
| NR2F1 | 7025 | nuclear receptor subfamily 2, group F, member 1 |
| NRCAM | 4897 | neuronal cell adhesion molecule |
| NRG1 | 3084 | neuregulin 1 |
| NRP1 | 8829 | neuropilin 1 |
| NT5E | 4907 | 5'-nucleotidase, ecto (CD73) |
| OAS1 | 4938 | 2',5'-oligoadenylate synthetase 1, 40/46kDa |
| OCLN | 4950 | occludin |
| OLFML2A | 169611 | olfactomedin-like 2A |
| OSR2 | 116039 | odd-skipped related 2 (Drosophila) |
| OVOL2 | 58495 | ovo-like 2 (Drosophila) |
| PAPSS2 | 9060 | 3'-phosphoadenosine 5'-phosphosulfate synthase 2 |
| PCDH7 | 5099 | protocadherin 7 |
| PDE4DIP | 9659 | phosphodiesterase 4D interacting protein |
| PDGFRL | 5157 | platelet-derived growth factor receptor-like |
| PEG10 | 23089 | paternally expressed 10 |
| PI3 | 5266 | peptidase inhibitor 3, skin-derived |
| PKP2 | 5318 | plakophilin 2 |
| PKP3 | 11187 | plakophilin 3 |
| PLA2G16 | 11145 | phospholipase A2, group XVI |
| PLA2G4A | 5321 | phospholipase A2, group IVA (cytosolic, calcium-dependent) |
| PLAT | 5327 | plasminogen activator, tissue |
| PLAU | 5328 | plasminogen activator, urokinase |
| PLAUR | 5329 | plasminogen activator, urokinase receptor |
| PLCB4 | 5332 | phospholipase C, beta 4 |
| PLS1 | 5357 | plastin 1 |
| PLXNB1 | 5364 | plexin B1 |
| PMP22 | 5376 | peripheral myelin protein 22 |
| POLR3G | 10622 | polymerase (RNA) III (DNA directed) polypeptide G (32kD) |
| POSTN | 10631 | periostin, osteoblast specific factor |
| PPAP2B | 8613 | phosphatidic acid phosphatase type 2B |
| PPARG | 5468 | peroxisome proliferator-activated receptor gamma |
| PPL | 5493 | periplakin |
| PRKCA | 5578 | protein kinase C, alpha |
| PRRG4 | 79056 | proline rich Gla (G-carboxyglutamic acid) 4 (transmembrane) |
| PRSS23 | 11098 | protease, serine, 23 |
| PRSS8 | 5652 | protease, serine, 8 |
| PRUNE2 | 158471 | prune homolog 2 (Drosophila) |
| PTGER2 | 5732 | prostaglandin E receptor 2 (subtype EP2), 53kDa |
| PTGER4 | 5734 | prostaglandin E receptor 4 (subtype EP4) |
| PTGES | 9536 | prostaglandin E synthase |
| PTGFR | 5737 | prostaglandin F receptor (FP) |
| PTHLH | 5744 | parathyroid hormone-like hormone |
| PTX3 | 5806 | pentraxin 3, long |
| RAPGEF5 | 9771 | Rap guanine nucleotide exchange factor (GEF) 5 |
| RASSF6 | 166824 | Ras association (RalGDS/AF-6) domain family member 6 |
| RCBTB2 | 1102 | regulator of chromosome condensation (RCC1) and BTB (POZ) domain containing protein 2 |
| RECK | 8434 | reversion-inducing-cysteine-rich protein with kazal motifs |
| RGL1 | 23179 | ral guanine nucleotide dissociation stimulator-like 1 |
| RGS4 | 5999 | regulator of G-protein signaling 4 |
| RHOBTB3 | 22836 | Rho-related BTB domain containing 3 |
| RHOD | 29984 | ras homolog gene family, member D |
| ROR1 | 4919 | receptor tyrosine kinase-like orphan receptor 1 |
| S100A2 | 6273 | S100 calcium binding protein A2 |
| S100A8 | 6279 | S100 calcium binding protein A8 |
| S100A9 | 6280 | S100 calcium binding protein A9 |
| S100P | 6286 | S100 calcium binding protein P |
| SCNN1A | 6337 | sodium channel, nonvoltage-gated 1 alpha |
| SEL1L3 | 23231 | sel-1 suppressor of lin-12-like 3 (C. elegans) |
| SEMA3A | 10371 | sema domain, immunoglobulin domain (Ig), short basic domain, secreted, (semaphorin) 3A |
| SEMA5A | 9037 | sema domain, seven thrombospondin repeats (type 1 and type 1-like), transmembrane domain (TM) and short cytoplasmic domain, (semaphorin) 5A |
| SERPINA1 | 5265 | serpin peptidase inhibitor, clade A (alpha-1 antiproteinase, antitrypsin), member 1 |
| SERPINA3 | 12 | serpin peptidase inhibitor, clade A (alpha-1 antiproteinase, antitrypsin), member 3 |
| SERPINB1 | 1992 | serpin peptidase inhibitor, clade B (ovalbumin), member 1 |
| SERPINB2 | 5055 | serpin peptidase inhibitor, clade B (ovalbumin), member 2 |
| SERPINB7 | 8710 | serpin peptidase inhibitor, clade B (ovalbumin), member 7 |
| SERPINE1 | 5054 | serpin peptidase inhibitor, clade E (nexin, plasminogen activator inhibitor type 1), member 1 |
| SERPINE2 | 5270 | serpin peptidase inhibitor, clade E (nexin, plasminogen activator inhibitor type 1), member 2 |
| SFN | 2810 | stratifin |
| SFRP1 | 6422 | secreted frizzled-related protein 1 |
| SIRPA | 140885 | signal-regulatory protein alpha |
| SLC22A4 | 6583 | solute carrier family 22 (organic cation/ergothioneine transporter), member 4 |
| SLC27A2 | 11001 | solute carrier family 27 (fatty acid transporter), member 2 |
| SLC39A8 | 64116 | solute carrier family 39 (zinc transporter), member 8 |
| SLC4A7 | 9497 | solute carrier family 4, sodium bicarbonate cotransporter, member 7 |
| SLC6A15 | 55117 | solute carrier family 6 (neutral amino acid transporter), member 15 |
| SLC7A5 | 8140 | solute carrier family 7 (cationic amino acid transporter, y+ system), member 5 |
| SLPI | 6590 | secretory leukocyte peptidase inhibitor |
| SMAD3 | 4088 | SMAD family member 3 |
| SMPDL3B | 27293 | sphingomyelin phosphodiesterase, acid-like 3B |
| SOCS2 | 8835 | suppressor of cytokine signaling 2 |
| SORL1 | 6653 | sortilin-related receptor, L(DLR class) A repeats containing |
| SPARC | 6678 | secreted protein, acidic, cysteine-rich (osteonectin) |
| SPINT1 | 6692 | serine peptidase inhibitor, Kunitz type 1 |
| SPOCK1 | 6695 | sparc/osteonectin, cwcv and kazal-like domains proteoglycan (testican) 1 |
| SPRR1A | 6698 | small proline-rich protein 1A |
| SPRY1 | 10252 | sprouty homolog 1, antagonist of FGF signaling (Drosophila) |
| SRCAP | 10847 | Snf2-related CREBBP activator protein |
| SRGN | 5552 | serglycin |
| SRPX2 | 27286 | sushi-repeat containing protein, X-linked 2 |
| ST14 | 6768 | suppression of tumorigenicity 14 (colon carcinoma) |
| ST6GALNAC2 | 10610 | ST6 (alpha-N-acetyl-neuraminyl-2,3-beta-galactosyl-1,3)-N-acetylgalactosaminide alpha-2,6-sialyltransferase 2 |
| STARD13 | 90627 | StAR-related lipid transfer (START) domain containing 13 |
| STC1 | 6781 | stanniocalcin 1 |
| SULF1 | 23213 | sulfatase 1 |
| SYK | 6850 | spleen tyrosine kinase |
| SYNE1 | 23345 | spectrin repeat containing, nuclear envelope 1 |
| SYT11 | 23208 | synaptotagmin XI |
| SYTL2 | 54843 | synaptotagmin-like 2 |
| TAGLN | 6876 | transgelin |
| TBX3 | 6926 | T-box 3 |
| TCF4 | 6925 | transcription factor 4 |
| TFPI | 7035 | tissue factor pathway inhibitor (lipoprotein-associated coagulation inhibitor) |
| TFPI2 | 7980 | tissue factor pathway inhibitor 2 |
| TGFA | 7039 | transforming growth factor, alpha |
| TGFBR3 | 7049 | transforming growth factor, beta receptor III |
| TGM2 | 7052 | transglutaminase 2 (C polypeptide, protein-glutamine-gamma-glutamyltransferase) |
| THBS1 | 7057 | thrombospondin 1 |
| TIMP2 | 7077 | TIMP metallopeptidase inhibitor 2 |
| TLR3 | 7098 | toll-like receptor 3 |
| TM4SF1 | 4071 | transmembrane 4 L six family member 1 |
| TMEM158 | 25907 | transmembrane protein 158 (gene/pseudogene) |
| TMEM30B | 161291 | transmembrane protein 30B |
| TNC | 3371 | tenascin C |
| TNFAIP3 | 7128 | tumor necrosis factor, alpha-induced protein 3 |
| TNFAIP6 | 7130 | tumor necrosis factor, alpha-induced protein 6 |
| TP63 | 8626 | tumor protein p63 |
| TPD52L1 | 7164 | tumor protein D52-like 1 |
| TPM1 | 7168 | tropomyosin 1 (alpha) |
| TPM2 | 7169 | tropomyosin 2 (beta) |
| TRIM29 | 23650 | tripartite motif containing 29 |
| TSPAN1 | 10103 | tetraspanin 1 |
| TUBA1A | 7846 | tubulin, alpha 1a |
| TXNIP | 10628 | thioredoxin interacting protein |
| VCAN | 1462 | versican |
| VEGFA | 7422 | vascular endothelial growth factor A |
| VGLL1 | 51442 | vestigial like 1 (Drosophila) |
| VIM | 7431 | vimentin |
| VSNL1 | 7447 | visinin-like 1 |
| WNT5A | 7474 | wingless-type MMTV integration site family, member 5A |
| XYLT1 | 64131 | xylosyltransferase I |
| ZEB1 | 6935 | zinc finger E-box binding homeobox 1 |
| ZHX2 | 22882 | zinc fingers and homeoboxes 2 |
| ZNF165 | 7718 | zinc finger protein 165 |
