# Supplementary material for: Meta-Analysis of Gene Expression Signatures Defining the Epithelial to Mesenchymal Transition during Cancer Progression
Source: PLoS One. 2012 Dec 10;7(12):e51136. doi: 10.1371/journal.pone.0051136 (PMC3519484; doi:10.1371/journal.pone.0051136)
Supplement: Table S3 — EMT-core gene list of 130 up- or downregulated genes shared between at least 10 GES datasets. (DOC) [file pone.0051136.s006.doc]

**Table S3:** EMT-core gene list of 130 up- or downregulated genes shared between at least 10 GES datasets.

| **Symbol** | **EntrezID** | **Name** | **Other categories** |
| --- | --- | --- | --- |
| **Upregulated genes** | | | |
| *a) Cell adhesion and migration* | | | |
| ADAM12 | 8038 | ATP-binding cassette, sub-family A (ABC1), member 1 | b |
| CDH11 | 1009 | cadherin 11, type 2, OB-cadherin (osteoblast) | b |
| CDH2 | 1000 | cadherin 2, type 1, N-cadherin (neuronal) | b |
| COL1A1 | 1277 | collagen, type I, alpha 1 | b |
| COL3A1 | 1281 | collagen, type III, alpha 1 | b |
| COL5A1 | 1289 | collagen, type V, alpha 1 | b |
| COL6A1 | 1291 | collagen, type VI, alpha 1 | b |
| COL6A3 | 1293 | collagen, type VI, alpha 3 | b |
| CTGF | 1490 | connective tissue growth factor | b, c, d, e |
| CYP1B1 | 1545 | cytochrome P450, family 1, subfamily B, polypeptide 1 | b, d |
| DLC1 | 10395 | deleted in liver cancer 1 | b |
| FBLN1 | 2192 | fibulin 1 | b |
| FBLN5 | 10516 | fibulin5 | b |
| FGF2 | 2247 | fibroblast growth factor 2 (basic) | b, c, d, e |
| FGFR1 | 2260 | fibroblast growth factor receptor 1 | b, c, e |
| FN1 | 2335 | fibronectin 1 | b, c, e |
| HAS2 | 3037 | hyaluronan synthase 2 | b |
| LAMC2 | 3918 | laminin, gamma 2 |  |
| LUM | 4060 | lumican | b |
| MMP2 | 4313 | matrix metallopeptidase 2 (gelatinase A, 72kDa gelatinase, 72kDa type IV collagenase) | b, c, e |
| MYL9 | 10398 | myosin, light chain 9, regulatory | b |
| NID2 | 22795 | nidogen 2 (osteonidogen) |  |
| NR2F1 | 7025 | nuclear receptor subfamily 2, group F, member 1 | b, d |
| NRP1 | 8829 | neuropilin 1 | b, c |
| PLAT | 5327 | plasminogen activator, tissue | b, c |
| PPAP2B | 8613 | phosphatidic acid phosphatase type 2B | b, c |
| PRKCA | 5578 | protein kinase C, alpha | b, c, d, e |
| RECK | 7434 | reversion-inducing-cysteine-rich protein with kazal motifs | b, c |
| SERPINE1 | 5054 | serpin peptidase inhibitor, clade E, member 1 | b, c, e |
| SERPINE2 | 5270 | serpin peptidase inhibitor, clade E, member 2 | b, c |
| SPOCK1 | 6695 | sparc/osteonectin, cwcv and kazal-like domains proteoglycan (testican) 1 | b |
| TGM2 | 7052 | transglutaminase | b, c, e |
| TNFAIP6 | 7130 | tumor necrosis factor, alpha-induced protein 6 | b, c |
| TPM1 | 7168 | tropomyosin 1 (alpha) | b, c |
| VCAN | 1462 | versican | b, c |
| WNT5A | 7474 | wingless-type MMTV integration site family, member 5A | b, c, e |
| *b) Development / cell differentiation and proliferation* | | | |
| CDKN2C | 1031 | cyclin-dependent kinase inhibitor 2C (p18, inhibits CDK4) | d, e |
| EMP3 | 2014 | epithelial membrane protein 3 | d, e |
| FBN1 | 2200 | fibrillin 1 |  |
| IGFBP3 | 3486 | insulin-like growth factor binding protein 3 | d, e |
| IL1R1 | 3554 | interleukin 1 receptor, type I |  |
| LTBP1 | 4052 | latent transforming growth factor beta binding protein 1 |  |
| MME | 4311 | membrane metallo-endopeptidase | d |
| PMP22 | 5376 | peripheral myelin protein 22 |  |
| PTGER2 | 5732 | prostaglandin E receptor 2 (subtype EP2), 53kDa |  |
| PTX3 | 5806 | pentraxin 3, long | c, d |
| SRGN | 5552 | serglycin | c, e |
| SULF | 23213 | sulfatase 1 | c |
| SYNE1 | 23345 | spectrin repeat containing, nuclear envelope 1 | e |
| TAGLN | 6876 | transgelin |  |
| TUBA1A | 7846 | tubulin, alpha 1a |  |
| VIM | 7431 | vimentin | e |
| ZEB1 | 6935 | zinc finger E-box binding homeobox 1 |  |
| *c) Angiogenesis and wound healing* | | | |
| DCN | 1634 | decorin | a, b, e |
| LOX | 4015 | lysyl oxidase | a, b, d |
| TFPI | 7035 | tissue factor pathway inhibitor (lipoprotein-associated coagulation inhibitor) |  |
| *d) Metabolism* | | |  |
| ABCA1 | 19 | ATP-binding cassette, sub-family A (ABC1), member 1 | b |
| GALNT10 | 55568 | GalNAc-T10 |  |
| SLC22A4 | 6583 | solute carrier family 22 (organic cation/ergothioneine transporter), member 4 |  |
| *Others or unclassified* | | |  |
| C5orf13 | 9315 | chromosome 5 open reading frame 13 |  |
| CDK14 | 5218 | cyclin-dependent kinase 14 |  |
| EML1 | 2009 | echinoderm microtubule associated protein like 1 |  |
| FSTL1 | 11167 | follistatin-like 1 |  |
| LTBP2 | 4053 | latent transforming growth factor beta binding protein 2 |  |
| MAP1B | 4131 | microtubule-associated protein 1B |  |
| RGS4 | 5999 | regulator of G-protein signaling 4 |  |
| SYT11 | 23208 | synaptotagmin XI |  |
| TMEM158 | 25907 | transmembrane protein 158 (gene/pseudogene) |  |
| **Downregulated genes** | | | |
| *a) Cell adhesion and migration* | | | |
| CD24 | 100133941 | CD24 molecule | b, e |
| CDH1 | 999 | cadherin 1, type 1, E-cadherin (epithelial) | b, e |
| CXADR | 1525 | coxsackie virus and adenovirus receptor | b, c |
| CXCL16 | 58191 | chemokine (C-X-C motif) ligand 16 | b |
| DSG3 | 1830 | desmoglein 3 | e |
| ELF3 | 1999 | E74-like factor 3 (ets domain transcription factor, epithelial-specific ) | b, c, e |
| EPCAM | 4072 | epithelial cell adhesion molecule | b |
| EPHA1 | 2041 | EPH receptor A1 | b |
| JUP | 3728 | junction plakoglobin | b |
| MPZL2 | 10205 | myelin protein zero-like 2 | b |
| OVOL2 | 58495 | ovo-like 2 (Drosophila) | b |
| PLXNB1 | 5364 | plexin B1 | b |
| S100P | 6286 | S100 calcium binding protein P |  |
| SLC7A5 | 8140 | solute carrier family 7 (cationic amino acid transporter, y+ system), member 5 | b, c |
| SYK | 6850 | spleen tyrosine kinase | b, c, d |
| *b) Development / cell differentiation and proliferation* | | | |
| ABLIM1 | 3983 | actin binding LIM protein 1 | a |
| ADRB2 | 154 | adrenergic, beta-2-, receptor, surface | c, e |
| ALDH1A3 | 220 | aldehyde dehydrogenase 1 family, member A3 | e |
| ANK3 | 288 | ankyrin 3, node of Ranvier (ankyrin G) |  |
| BIK | 638 | BCL2-interacting killer (apoptosis-inducing) | e |
| CA2 | 760 | carbonic anhydrase II |  |
| CTSL2 | 1515 | cathepsin L2 | d, e |
| FGFR2 | 2263 | fibroblast growth factor receptor 2 |  |
| FGFR3 | 2261 | fibroblast growth factor receptor 3 |  |
| FST | 10468 | follistatin |  |
| GJB3 | 2707 | gap junction protein, beta 3, 31kDa |  |
| IFI30 | 10437 | interferon, gamma-inducible protein 30 |  |
| IL18 | 3606 | interleukin 18 (interferon-gamma-inducing factor) | a, c, e |
| KLK7 | 5650 | kallikrein-related peptidase 7 |  |
| KRT15 | 3866 | keratin 15 |  |
| KRT17 | 3872 | keratin 17 |  |
| LSR | 51599 | lipolysis stimulated lipoprotein receptor | d |
| MAP7 | 9053 | microtubule-associated protein 7 | d |
| MBP | 4155 | myelin basic protein |  |
| OCLN | 4950 | occludin | e |
| PKP2 | 5318 | plakophilin 2 | a |
| PPL | 5493 | periplakin |  |
| PRSS8 | 5652 | protease, serine, 8 | d |
| RAPGEF5 | 9771 | Rap guanine nucleotide exchange factor (GEF) 5 |  |
| SPINT1 | 6692 | serine peptidase inhibitor, Kunitz type 1 |  |
| *d) Metabolism* | | |  |
| GPX3 | 2878 | glutathione peroxidase 3 (plasma) | b |
| SLC27A2 | 11001 | solute carrier family 27 (fatty acid transporter), member 2 |  |
| SMPDL3B | 27293 | sphingomyelin phosphodiesterase, acid-like 3B |  |
| SORL1 | 6653 | sortilin-related receptor, L(DLR class) A repeats containing | a |
| ST6GALNAC2 | 10610 | ST6 -N-acetylgalactosaminide alpha-2,6-sialyltransferase 2 |  |
| *Others or unclassified* | | |  |
| AGR2 | 10551 | anterior gradient homolog 2 (Xenopus laevis) |  |
| C10orf10 | 11067 | chromosome 10 open reading frame 10 |  |
| CDS1 | 1040 | CDP-diacylglycerol synthase (phosphatidate cytidylyltransferase) 1 |  |
| FAM169A | 26049 | family with sequence similarity 169, member A |  |
| FXYD3 | 5349 | FXYD domain containing ion transport regulator 3 |  |
| KLK10 | 5655 | kallikrein-related peptidase 10 |  |
| LAD1 | 3898 | ladinin 1 |  |
| MTUS1 | 57509 | microtubule associated tumor suppressor 1 |  |
| PLS1 | 5357 | plastin 1 |  |
| PRRG4 | 79056 | proline rich Gla (G-carboxyglutamic acid) 4 (transmembrane) |  |
| RHOD | 29984 | ras homolog gene family, member D |  |
| SERPINB1 | 1992 | serpin peptidase inhibitor, clade B (ovalbumin), member 1 |  |
| SLPI | 6590 | secretory leukocyte peptidase inhibitor |  |
| TMEM30B | 161291 | transmembrane protein 30B |  |
| TPD52L1 | 7164 | tumor protein D52-like 1 | e |
| TSPAN1 | 10103 | tetraspanin 1 |  |
| ZHX2 | 22882 | zinc fingers and homeoboxes 2 |  |
| ZNF165 | 7718 | zinc finger protein 165 |  |

Categories have been chosen according to the GO classifications obtained by enrichment tools. Genes may be present in more than one category.

a, cell adhesion/migration; b, development or cell differentiation/proliferation; c, angiogenesis/wound healing; d, metabolism; e, apoptosis.
