# Supplementary material for: Meta-Analysis of Gene Expression Signatures Defining the Epithelial to Mesenchymal Transition during Cancer Progression
Source: PLoS One. 2012 Dec 10;7(12):e51136. doi: 10.1371/journal.pone.0051136 (PMC3519484; doi:10.1371/journal.pone.0051136)
Supplement: Table S4 — Enrichment tools used in this study and their properties. (DOC) [file pone.0051136.s007.doc]

**Table S4:** Enrichment tools used in this study and their properties.

| **Tool** | **First ref.** | **Key statistical method** | **Multiple testing correction method(s)** |
| --- | --- | --- | --- |
| ConsensusPathDB* | [1] | Hypergeometric | FDR |
| FatiGO | [2] | Fisher’s exact | 3 methods (B-H, B-Y, permutation) |
| GeneCodis | [3] | Hypergeometric | FDR^#^, permutation |
| ToppFun | [4] | Hypergeometric | Bonferroni, FDR^#^ |
| WebGestalt | [5] | Hypergeometric | B-H^#^, B-Y, Bonferroni, holm, hommel |

*, uses gene ontology levels 2-4 only; ^#^, indicates the multiple testing method which was used.

References to Table S4:

1. Kamburov A, Wierling C, Lehrach H, Herwig R (2009) ConsensusPathDB--a database for integrating human functional interaction networks. Nucleic Acids Res 37: D623-628.

2. Al-Shahrour F, Diaz-Uriarte R, Dopazo J (2004) FatiGO: a web tool for finding significant associations of Gene Ontology terms with groups of genes. Bioinformatics 20: 578-580.

3. Carmona-Saez P, Chagoyen M, Tirado F, Carazo JM, Pascual-Montano A (2007) GENECODIS: a web-based tool for finding significant concurrent annotations in gene lists. Genome Biol 8: R3.

4. Chen J, Bardes EE, Aronow BJ, Jegga AG (2009) ToppGene Suite for gene list enrichment analysis and candidate gene prioritization. Nucleic Acids Res 37: W305-311.

5. Zhang B, Kirov S, Snoddy J (2005) WebGestalt: an integrated system for exploring gene sets in various biological contexts. Nucleic Acids Res 33: W741-748.
